# Respiratory syncytial and influenza viruses in children under 2 years old with severe acute respiratory infection (SARI) in Maputo, 2015

**DOI:** 10.1371/journal.pone.0186735

**Published:** 2017-11-30

**Authors:** Mirela Pale, Afonso Nacoto, Almiro Tivane, Neuza Nguenha, Loira Machalele, Félix Gundane, Délcio Muteto, Josina Chilundo, Sandra Mavale, Cynthia Semá-Baltazar, Germano Pires, Orvalho Augusto, Tufária Mussá, Eduardo Gudo

**Affiliations:** 1 Department of Technologic Platforms, National Institute of Health, Ministry of Health, Maputo, Mozambique; 2 Pediatric Departments, Maputo Central Hospital, Maputo, Mozambique; 3 Department of Microbiology, Faculty of Medicine, Eduardo Mondlane University, Maputo, Mozambique; University of Tennessee Health Science Center, UNITED STATES

## Abstract

**Introduction:**

Although respiratory syncytial virus (RSV) and influenza virus (influenza) infections are one of the leading causes of Severe Acute Respiratory Infections (SARI) and death in young children worldwide, little is known about the burden of these pathogens in Mozambique.

**Material and methods:**

From January 2015 to January 2016, nasopharyngeal swabs from 450 children, aged ≤2 years, who had been admitted to the Pediatric Department of the Maputo Central Hospital (HCM) in Mozambique, suffering with SARI were enrolled and tested for influenza and RSV using a real-time PCR assay.

**Results:**

Influenza and RSV were detected in 2.4% (11/450) and 26.7% (113/424) of the participants. Children with influenza were slightly older than those infected with RSV (10 months in influenza-infected children compared to 3 months in RSV-infected children); male children were predominant in both groups (63.6% versus 54.9% in children with influenza and RSV, respectively). There was a trend towards a higher frequency of influenza (72.7%) and RSV (93.8%) cases in the dry season. Bronchopneumonia, bronchitis and respiratory distress were the most common diagnoses at admission. Antibiotics were administered to 27,3% and 15,9% of the children with influenza and RSV, respectively. Two children, of whom, one was positive for RSV (aged 6 months) and another was positive for Influenza (aged 3 months) died; both were children of HIV seropositive mothers and had bronchopneumonia.

**Conclusions:**

Our data demonstrated that RSV, and less frequently influenza, occurs in children with SARI in urban/sub-urban settings from southern Mozambique. The occurrence of deaths in small children suspected of being HIV-infected, suggests that particular attention should be given to this vulnerable population. Our data also provide evidence of antibiotics prescription in children with respiratory viral infection, which represents an important public health problem and calls for urgent interventions.

## Introduction

Acute lower respiratory tract infections (ARI) are the leading cause of childhood morbidity and pediatric death worldwide [1–3], accounting for an estimated 1.9 million deaths annually in children under-five years of age. Of these deaths, up to 90% are known to occur in developing countries [4–6] and almost 50% occur in sub-Saharan Africa [4, 7]. The severe form of ARI, known as Severe Acute Respiratory Illness (SARI) is responsible for an estimated 20% of deaths in children under-five years of age worldwide [4, 7, 8].

With the introduction of vaccination against *S*. *pneumoniae* and *H*. *influenzae*, viruses become the major cause of SARIs in children younger than 5 years [9], with Influenza virus (influenza) and Respiratory Syncytial virus (RSV) accounting for a substantial proportion of hospital admission and deaths attributed to SARI at this age [1, 5, 6]. It is estimated that seasonal influenza affects 5–10% of the world population annually, resulting in as many as 110 000 influenza-associated deaths in children <5 years of age [5, 10]. On the other hand, RSV is known to be most important cause of SARI in young children worldwide [6, 9, 11, 12], and the leading cause of pneumonia and bronchiolitis in infants and children resulting in as many as 33.8 million hospitalizations and 66 000 to199 000 deaths each year around the globe [6, 13].

Information on the viral etiology of ARI/SARI in Mozambique is very limited and the few studies conducted in the country to date are from a geographically limited rural district situated in southern Mozambique [14–16]. No data exist about these infections in urban and suburban areas, where population density and overcrowded environments are increasing rapidly. Of note, all studies were conducted prior to the introduction of the PCV10 vaccine into the national immunization schedule.

In Mozambique, where under-five mortality rate is estimated at 78.5 per 100,000 live births, there is renewed public health interest in SARI, influenza and RSV in children because recent data from South Africa have shown that HIV-infected children are at particularly high risk of developing SARI and death [17, 18]. Since Mozambique has the eighth highest HIV prevalence in the world [19, 20], where an estimated 110,000 children are living with HIV, we anticipate that rates and consequences of SARI are high. This study was conducted to investigate the epidemiological and clinical features of influenza and RSV among children ≤2 years of age admitted with SARI to the Pediatric Department of Maputo Central Hospital from January 2015 through January 2016.

## Material and methods

### Ethic statement

The SARI surveillance protocol was approved by the National Bioethics Committee for Health (**IBR:** 00002657, study ref: 172/CNBS/2014). Only verbal consent was obtained in order to minimize significant interference in the routine care being provided to each child at the sentinel sites. Our surveillance was embedded into the routine medical care. We used a log book to record all child whose legal guardian consented to participate.

### Study setting and participants

We conducted a hospital-based surveillance study among children ≤2 years admitted to the breastfeeding and respiratory disease wards of the Maputo Central Hospital (MCH) suffering with SARI, from January 2015 to January 2016. The MCH is a quaternary hospital located in Maputo city, the capital of the country in southern Mozambique. The Pediatric Department of the MCH has a total of 324 beds. The hospital is a national reference hospital for the entire country and also serves a large population living in the urban and suburban districts of Maputo city. A total of 1.782.380 inhabitants lives in Maputo city, distributed over a total of 346.77 km^2^. The climate in Maputo is tropical with two seasons, namely, rainy season from November to April and the dry season during the rest of the year. Poverty, poor sanitation, low education level, and overcrowding characterize the sub-urban area of Maputo city. The main source of income in this area is informal trade and small business.

### Case definition

As per World Health Organization, a case of SARI was defined as any severe patient (requiring hospitalization) with acute symptoms (within the last 10 days of onset of disease); and respiratory infection (defined as the presence of cough, but in some sites defined as cough or shortness of breath).

### Enrolment and sample collection

Each day, during the recruitment period, the first 3 children aged between 1 month and ≤ 2 years who met the SARI case definition were recruited. During this study (January 2015 to January 2016), a total of 775 children were admitted with SARI to the breastfeeding and respiratory disease wards of the Maputo Central Hospital, of whom 450 (58.1%) were enrolled in this study. Verbal consent was obtained from their parent or legal guardian, prior to data and specimen collection. Demographic, clinical and epidemiological information was collected by trained nurses or physicians using a case report form. In addition, nasopharyngeal swabs were collected from all enrolled children. Swabs were then placed in a small tube containing universal transport medium and preserved at 4–8°C. Samples were sent daily to the Virus Isolation Laboratory (VIL) at the National Institute of Health for molecular testing.

### Laboratory procedures

Upon receipt at the VIL, samples were aliquoted and stored at -70°C. RNA was extracted using the *QIAamp RNA Viral Mini Kit* (QUIAGEN Inc., CA) following the manufacturer instructions.

For detection and typing of influenza virus, a real-time reverse-transcriptase polymerase chain reaction assay (RT-PCR) was carried out using the human influenza virus real-time RT-PCR diagnostic panel and a protocol developed and kindly provided by the US Centers for Disease Control and Prevention (CDC, USA) (S1 File). For RSV detection, the Fast-track Diagnostics Respiratory pathogens 2 plus (FTD 2 Plus, Luxembourg) kit was used following the manufacturer’s instructions. Only influenza-negative samples were tested for RSV.

**Fig 1:**
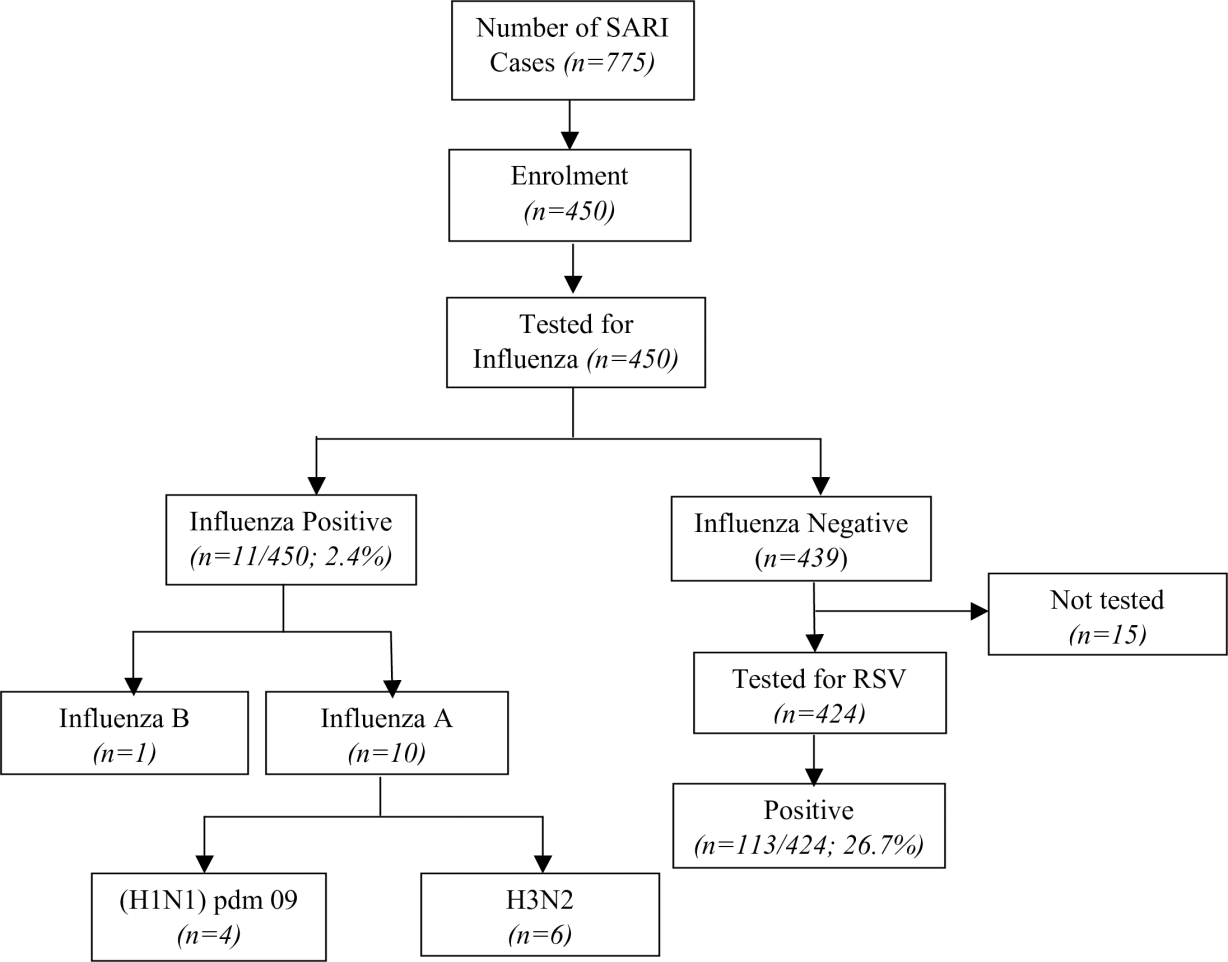
Flowchart of recruitment of study participants and sample testing. Of the 439 negative samples for influenza, 424 were tested for RSV; 15 samples were not tested due to lack of reagents.

Samples from 450 participants were tested for influenza. All influenza negative samples (n = 439) were subsequently tested for RSV, although 15 of them were not tested for RSV due to lack of reagents, thus the total number of samples tested for RSV was 424 (Fig 1).

### Statistical analysis

Data were entered into a database developed using Microsoft Office Access 2010 software (Microsoft corporation, USA) and analyzed using the SPSS version 20 (IBM Corp., Armonk, NY, USA). The *X*^*2*^ test was used to compare frequencies and Mann-Whitney was used to compare medians.

## Results

### General characteristics of enrolled children

From January 2015 to January 2016, a total of 450 children ≤2 years old with SARI were enrolled. The median age of children with SARI was 6 months (IQR: 2–13) and 51.5% (230/450) were male. Most of the patients with SARI were enrolled during the dry season (67.3%; 303/450). At admission, the main symptoms of children with SARI were cough (91.3%), followed by dyspnoea (73.6%) and rhinorrhea (60.4%). The main form of presentation of SARI at admission was bronchopneumonia (46.0%; 207/450), followed by bronchitis (29.3%; 132/450) and respiratory distress (24.4%; 110/450). Asthma was reported in 13.3% (60/450). of children with SARI.

Treatment with antibiotics was administered to 34.9% (157/450) of children with SARI and oxygen was given to 4.2% (19/450) of them (Table 1 and S1 Table).

**Table 1:** Clinical and demographic characteristics of participants.

	Influenza			RSV		
Characteristic	Tested N (%)	Positive N (%)	Prevalence* % (95CI)	Tested N (%)	Positive N (%)	Prevalence* % (95CI)
**Total**	450 (100.0)	11 (100.0)	2.4 (1.2–4.3)	424 (100.0)	113 (100.0)	26.7 (22.5–31.1)
**Age in months**						
Min–Max	0.3–23	2–21		0.3–23	1–23	
Median (IQR)	6 (2–13)	10 (3–18)		6 (2–13)	3 (2–8)	
** Categories**						
< 3	131 (29.1)	2 (18.2)	1.5 (0.2–5.4)	127 (30.0)	55 (48.7)	43.3 (34.5–52.4)
3–5	72 (16.0)	1 (9.1)	1.4 (0.0–7.5)	67 (15.8)	19 (16.8)	28.4 (18.0–40.7)
6–11	121 (26.9)	3 (27.3)	2.5 (0.5–7.1)	114 (26.9)	19 (16.8)	16.7 (10.3–24.8)
12–23	126 (28.0)	5 (45.5)	4.0 (1.3–9.0)	116 (27.4)	20 (17.7)	17.2 (10.9–25.4)
**Gender**						
Male	230 (51.1)	7 (63.6)	3.0 (1.2–6.2)	220 (51.9)	62 (54.9)	28.2 (22.3–34.6)
Female	202 (44.9)	4 (36.4)	2.0 (0.5–5.0)	186 (43.9)	43 (38.1)	23.1 (17.3–29.8)
No information	18 (4.0)	0 (0.0)	0.0 (< 18.5)	18 (4.2)	8 (7.1)	44.4 (21.5–69.2)
**Season**						
Dry season	303 (67.3)	8 (72.7)	2.6 (1.1–5.1)	295 (69.6)	106 (93.8)	35.9 (30.5–41.7)
Wet season	147 (32.7)	3 (27.3)	2.0 (0.4–5.8)	129 (30.4)	7 (6.2)	5.4 (2.2–10.9)
**Signs or symptoms**						
Cough	411 (91.3)	10 (90.9)	2.4 (1.2–4.4)	388 (91.5)	107 (94.7)	27.6 (23.2–32.3)
Dyspnea	331 (73.6)	8 (72.7)	2.4 (1.0–4.7)	313 (73.8)	93 (82.3)	29.7 (24.7–35.1)
Rhinorrhea	272 (60.4)	4 (36.4)	1.5 (0.4–3.7)	262 (61.8)	81 (71.7)	30.9 (25.4–36.9)
Fever	178 (39.6)	7 (63.6)	3.9 (1.6–7.9)	171 (40.3)	56 (49.6)	32.7 (25.8–40.3)
Cough and Dyspnea and Fever	137 (30.4)	5 (45.5)	3.6 (1.2–8.3)	132 (31.1)	50 (44.2)	37.9 (29.6–46.7)
All 4 symptoms	97 (21.6)	4 (36.4)	4.1 (1.1–10.2)	93 (21.9)	38 (33.6)	40.9 (30.8–51.5)
**Diagnoses at admission**						
Bronchopneumonia	207 (46.0)	5 (45.5)	2.4 (0.8–5.5)	196 (46.2)	50 (44.2)	25.5 (19.6–32.2)
Pneumonia	41 (9.1)	0 (0.0)	0.0 (< 8.6)	38 (9.0)	6 (5.3)	15.8 (6.0–31.3)
Bronchitis	132 (29.3)	2 (18.2)	1.5 (0.2–5.4)	128 (30.2)	45 (39.8)	35.2 (26.9–44.1)
Respiratory distress	110 (24.4)	2 (18.2)	1.8 (0.2–6.4)	101 (23.8)	27 (23.9)	26.7 (18.4–36.5)
Asthma	60 (13.3)	1 (9.1)	1.7 (0.0–8.9)	55 (13.0)	6 (5.3)	10.9 (4.1–22.2)
URTI	22 (4.9)	2 (18.2)	9.1 (1.1–29.2)	18 (4.2)	6 (5.3)	33.3 (13.3–59.0)
Previous cardio-respiratory chronic disease	9 (2.0)	0 (0.0)	0.0 (< 33.6)	9 (2.1)	2 (1.8)	22.2 (2.8–60.0)
**Medical procedures**						
Antibiotics	157 (34.9)	3 (27.3)	1.9 (0.4–5.5)	148 (34.9)	18 (15.9)	12.3 (7.4–18.5)
Oxygenation	19 (4.2)	2 (18.2)	10.5 (1.3–33.1)	17 (4.0)	5 (4.4)	29.4 (10.3–56.0)
**Others medical procedures	278 (61.8)	6 (54.5)	2.3 (0.8–4.6)	263 (62.0)	90 (79.6)	34.2 (28.5–40.3)

*Prevalence is the positives column divided by the total column.

95CI– 95% confidence interval. These were computed through binomial exact method.

**Others medical procedures: Mechanical ventilation and admission to intensive care unit.

### Clinical and demographics characteristics of children with influenza or RSV

Frequencies of influenza and RSV in children with SARI were 2.4% (11/450) and 26.7% (113/424), respectively. The higher frequency of influenza cases was reported among children aged between 12–23 months (4.0%; 5/126), while the higher frequency of RSV was noted among children aged ≤3 months (43.3%; 55/127) (Table 1).

Children with influenza were slightly older than those infected with RSV, [10 months (IQR: 3–18) compared 3 months (IQR: 2–8) respectively]. Male children were predominant in both groups (63.6%; 7/11 versus 54.9%; 62/113 in children with influenza and RSV, respectively).

In both groups, cough and dyspnea were the most common symptoms. However, fever was the third most common symptom in children with influenza, while rhinorrhoea was the third most common symptom among RSV infected children. Bronchopneumonia, bronchitis and respiratory distress were the most common diagnoses at admission for both influenza and RSV infection.

Antibiotics were administered to 27,3% (3/11) and 15,9% (18/113) of children with influenza and RSV, respectively. During this study, a total of two children died, of whom, one was positive for RSV (aged 6 months) and another was positive for Influenza (aged 3 months). We found that both children had HIV seropositive mothers, had bronchopneumonia, and were ≤ 6 months of age. Oxygen was administrated to 2 (18.2%) children with Influenza and 5 (4.4%) children with RSV.

### Seasonality of influenza and RSV and typing of influenza virus

**Fig 2:**
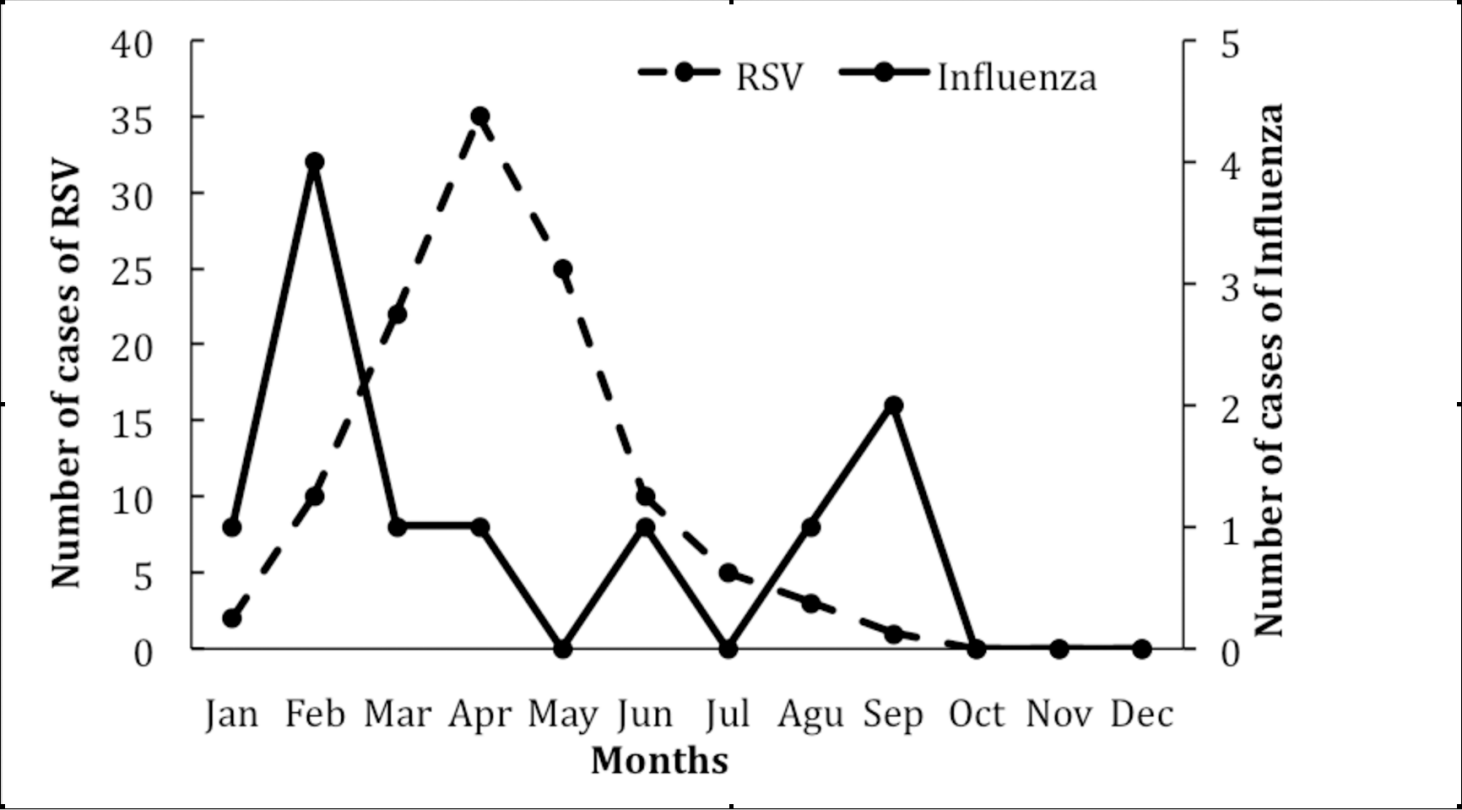
Monthly and seasonal variation of cases of influenza and RSV. The graph depicts the monthly and seasonal variation of cases of influenza (right axis) and RSV (left axis).

Although cases of Influenza and RSV occurred almost throughout the year, there was a trend towards the higher frequency of cases of influenza (72.7%; 8/11) and RSV (93.8%; 106/113) during the dry season (Table 1). On the other hand, while RSV occurred in the first semester of 2015, peaking in April, influenza showed a bimodal shape curve, occurring in the rainy and dry seasons (Fig 2). The most common types of Influenza were A (H3N2) (6/11; 54.5%), followed by type A (H1N1) pdm09 (4/11; 36.4%) and type B (1/11; 9.1%).

## Discussion

In Mozambique and many other countries in sub-Saharan Africa, there is a lack of data on the epidemiology and burden of influenza and RSV. Consequently, most of SARI cases are presumptively diagnosed as bacterial infections and treated with antibiotics [21]. We found a frequency of influenza and RSV among children admitted with SARI of 2.4% and 26.7%, respectively. Our results are important because, to the best of our knowledge, this is the first description of the burden of influenza and RSV in children suffering with SARI living in urban/sub-urban settings in Mozambique. The few studies that had been conducted in Mozambique to date were all from a small rural village situated in Southern Mozambique [14–16]. On the other hand, much has changed since these studies were conducted, such as the introduction of *Haemophilus influenza type b* and *S*. *pneumonia* vaccination in Mozambique in 2009 and 2013, respectively. In addition, the some of the underlying and predisposing factors such as the epidemiology of HIV and under-nutrition, varies in different regions of the country. Prevalence of RSV in this study was similar to that reported in a previous study conducted in a rural district in southern Mozambique [16], as well as in other countries in the region [17, 18, 22]. The prevalence of RSV in this study is consistent with current evidence that RSV is the single most frequent lower respiratory tract infection pathogen in infants and young children worldwide [6, 23–25]. However, other countries reported either higher [26, 27] or lower [23, 28, 29] prevalence than that found in our study. In regard to influenza, although our prevalence was lower than that reported in many countries in the region [30, 31], similar prevalence rates were reported in other countries [26, 28]

We found that RSV was more common in younger children (aged ≤ 3 months), while Influenza was more common in children aged 12–23 months. These results are similar to those reported by Resch et al 2011 [32]. Previous studies from other sub-Saharan Africa countries found that, not only the frequency, but also the severity of RSV was higher in children of younger age [11, 22, 23, 26, 33–35]. In regard to influenza, other studies in sub-Saharan Africa also found that the virus was more common in children older than 12 months [22]. The higher frequency of RSV in children aged ≤ 3 months highlight that public health interventions should prioritize this group of children.

The frequency of influenza and RSV in male was slightly higher when compared to female, which is consistent with findings from previous studies [11, 36].

RSV and influenza showed a different pattern of seasonality. As such, while RSV occurred in the first quarter of 2015, peaking in April, influenza had a bimodal curve shape, occurring in the rainy and dry seasons. Studies conducted in other countries in sub-Saharan Africa showed similar patterns [17, 18, 22, 23].

Bronchopneumonia, followed by bronchiolitis, was the most common form of presentation of SARI, influenza and RSV, which is consistent with available data from other countries in sub-Saharan Africa and other regions [8, 26, 28]. Indeed, RSV has been considered the major cause of severe pneumonia and bronchiolitis worldwide [6, 37].

Two children, one positive for influenza (age: 3 months) and another positive for RSV (age: 6 months), died during the study period. Both were admitted with bronchopneumonia and were children of HIV seropositive mothers. Although their HIV status was unknown, we believe that they were also infected by HIV, assuming that in Mozambique, the vertical transmission of HIV is still significant [38]. It’s well known that hospitalization and case-fatality rates are significantly higher in HIV infected children with ARI [17, 18, 34, 39]. This is particularly concerning for Mozambique, where the prevalence of HIV is one of the highest in the world [19, 20]. Recent data from South Africa found that even after rapid scale up of antiretroviral treatment, mortality among HIV-infected children remains significantly high [17, 18].

Use of oxygen therapy and presence of underlying chronic diseases were not common in our study, which is differ from what was reported in previous studies [26, 36, 40]. The lower frequency of use of oxygen-therapy can be attributed to a lower severity of the disease or to an inadequate diagnosis of hypoxia in Influenza and RSV infected children. However, one of the deceased child who was positive to influenza (age: 3 months) was submitted to oxygen-therapy. On the other hand, antibiotics were massively administrated to Influenza and RSV positive children, increasing the risk for antimicrobial resistance as suggested by several authors [21, 41, 42].

Although our study provides valuable data about SARI in urban/suburban children in southern Mozambique, we acknowledge some limitations of our study. First, not all eligible children who met the case definitions were identified and enrolled, however, selection of participants was systematic. As such, each day, only the first 3 children who met the case definition were recruited in order to minimize selection bias. Second, we acknowledge that our study was not designed to properly assess the role of HIV in influenza and RSV severity and fatality. For this reason, we recommend that future studies should be properly designed to investigate this. Lastly, several case investigation forms were incomplete due to lack of data in the patient file and for this reason, data of several variables that are relevant for this study, such as prematurity and smoke at home were not collected.

## Conclusions

Our results show that RSV is the main cause of SARI among children ≤2 years old admitted to the breastfeeding and respiratory disease wards of the main hospital in Southern Mozambique. Moreover, RSV-associated deaths occurred among children aged 6 months with bronchopneumonia and presumably infected with HIV, suggesting that preventive and therapeutic interventions for SARI should prioritize younger children infected by HIV. Our data also provides evidence on the antibiotics prescription in children with viral etiology, which may represent a public health concern due to the increased risk of antibiotic resistance and calls for urgent interventions, such as improvement of algorithms for clinical management of SARI, availability of laboratory tests to discriminate bacterial and nonbacterial infections and increase of awareness among clinicians.

## Supporting information

S1 Table Antibiotics prescribed for the children admitted with SARI. *A Child may have received two antibiotics. The combination were 10 Crystalline penicillin+Gentamicin; 6 Ampicillin + Gentamicin; 2 Cotrimoxazol+Amoxicillin; 2 Crystalline penicillin+Cotrimoxazol; 1 Crystalline penicillin+Cotrimoxazol+Gentamicin and 1 Crystalline penicillin+Ampicilina** ** This child has started with Cristaline penincilin and physician had changed the prescription to ampicillin. n = number of children that have received antibiotics. N = Total number of tested children (450).
(DOC)

S1 File CDC protocol for typing and sub typing of influenza viruses. For the detection and typing of influenza virus, a real-time reverse-transcriptase polymerase chain reaction assay (RT-PCR) was carried out using the human influenza virus real-time RT-PCR diagnostic panel and a protocol developed and kindly provided by the US Centers for Disease Control and Prevention (CDC, USA).
(PDF)

S2 File Minimal data set used in this study. A hospital-based surveillance study among children ≤2 years admitted to the breastfeeding and respiratory disease wards of the Maputo Central Hospital (MCH) suffering with SARI, were enrolled from January 2015 to January 2016.
(XLS)
