# Supplementary material for: Respiratory syncytial and influenza viruses in children under 2 years old with severe acute respiratory infection (SARI) in Maputo, 2015
Source: PLoS One. 2017 Nov 30;12(11):e0186735. doi: 10.1371/journal.pone.0186735 (PMC5708764; doi:10.1371/journal.pone.0186735)
Supplement: S1 Table — *A Child may have received two antibiotics. The combination were 10 Crystalline penicillin+Gentamicin; 6 Ampicillin + Gentamicin; 2 Cotrimoxazol+Amoxicillin; 2 Crystalline penicillin+Cotrimoxazol; 1 Crystalline penicillin+Cotrimoxazol+Gentamicin and 1 Crystalline penicillin+Ampicilina** ** This child has started with Cristaline penincilin and physician had changed the prescription to ampicillin. n = number of children that have received antibiotics. N = Total number of tested children (450). (DOC) [file pone.0186735.s001.doc]

|  | **Number of antibiotics prescribed** | | | |
| --- | --- | --- | --- | --- |
|  | 1 | 2 | 3 | |
| **Percentage of prescriptions (n/N)** | 30.0 (135/450) | 4.67 (21/450) | 0.22 (1/450) | |
|  | | | | |
| **Types of antibiotics** | Total SARI Children (%) | Influenza Positive (%) | | RSV Positive (%) |
| Amoxicillin | 14 (7.78) | 0 (0) | | 1 (5.26) |
| Ampicillin | 9 (5.0) | 0 (0) | | 2 (10.53) |
| Ceftriaxone | 1 (0.56) | 0 (0) | | 0 (0) |
| Cotrimoxazol | 12 (6.67) | 0 (0) | | 1(5.26) |
| Crystalline penicillin | 124 (68.89) | 2 (66.67) | | 15 (78.95) |
| Gentamicin | 20 (11.11) | 1 (33.33) | | 0 (0) |
| **Total** | **180* (100)** | **3 (100))** | | **19* (100** |
