# Supplementary material for: Respiratory syncytial and influenza viruses in children under 2 years old with severe acute respiratory infection (SARI) in Maputo, 2015
Source: PLoS One. 2017 Nov 30;12(11):e0186735. doi: 10.1371/journal.pone.0186735 (PMC5708764; doi:10.1371/journal.pone.0186735)
Supplement: S1 File — For the detection and typing of influenza virus, a real-time reverse-transcriptase polymerase chain reaction assay (RT-PCR) was carried out using the human influenza virus real-time RT-PCR diagnostic panel and a protocol developed and kindly provided by the US Centers for Disease Control and Prevention (CDC, USA). (PDF) [file pone.0186735.s002.pdf]

CDC Human Influenza Virus Real-Time RT-PCR Diagnostic Panel (CDC Flu rRT-PCR Dx Panel)

Influenza A/B Typing Kit

Instructions for Use
Package Insert

Catalog # FluIVD03-1
1000 reactions

For *In-vitro* Diagnostic (IVD) Use

Rx only

Centers for Disease Control and Prevention
Influenza Division
1600 Clifton Rd NE
Atlanta GA 30333

Table of Contents

Intended Use 2
Summary and Explanation 2
Principles of the Procedure 5
Materials Required (Provided) 7
Materials Required (But Not Provided) 8
Warnings and Precautions 10
Reagent Storage, Handling, and Stability 11
Specimen Collection, Handling, and Storage 12
Specimen Referral to CDC 12
Reagent and Controls Preparation 13
General Preparation 15
Assay Setup 16
Interpretation of Results and Reporting 35
Influenza A/B Typing Assay Results Interpretation Guide 37
Standards-Based Electronic Laboratory Reporting for Influenza 38
Quality Control 39
Limitations 39
Performance Characteristics 41
Disposal 53
References 53
Contact Information, Ordering, and Product Support 55

Intended Use

The Influenza A/B Typing Kit contains reagents and controls of the CDC Human Influenza Virus Real-Time RT-PCR Diagnostic Panel and is intended for use in real-time RT-PCR (rRT-PCR) assays on an Applied Biosystems (ABI) 7500 Fast Dx Real-Time PCR instrument in conjunction with clinical and epidemiological information:

- For qualitative detection of influenza virus type A or B viral RNA in upper respiratory tract clinical specimens (including nasopharyngeal swabs [NPS], nasal swabs [NS], throat swabs [TS], nasal aspirates [NA], nasal washes [NW] and dual nasopharyngeal/throat swabs [NPS/TS]) and lower respiratory tract specimens (including bronchoalveolar lavage [BAL], bronchial wash [BW], tracheal aspirate [TA], sputum, and lung tissue) from human patients with signs and symptoms of respiratory infection and/or from viral culture.
- To provide epidemiologic information for surveillance of circulating influenza viruses.

Performance characteristics for influenza were established during a season when seasonal influenza viruses A/H1 and A/H3 were the predominant influenza A viruses in circulation and during a season when the A/H1pdm09 influenza virus was the predominant influenza A virus in circulation. Performance characteristics may vary with other emerging influenza A viruses.

Negative results do not preclude influenza virus infection and should not be used as the sole basis for treatment or other patient management decisions. Conversely, positive results do not rule out bacterial infection or co-infection with other viruses. The agent detected may not be the definite cause of disease.

If infection with a novel influenza A virus is suspected based on current clinical and epidemiological screening criteria recommended by public health authorities, specimens should be collected with appropriate infection control precautions for novel virulent influenza viruses and sent to state or local health department for testing. Viral culture should not be attempted in these cases unless a BSL 3+ facility is available to receive and culture specimens.

All users, analysts, and any person reporting results from use of this device should be trained to perform and interpret the results from this procedure by a competent instructor prior to use. CDC Influenza Division will limit the distribution of this device to only those users who have successfully completed a training course provided by CDC instructors or designees.

Summary and Explanation

Influenza viruses are the causative agents of influenza infection, an acute respiratory illness that is highly contagious and results in significant morbidity each year with flu-related deaths ranging from a low of about 3,000 to a high of about 49,000 people (http://www.cdc.gov/flu/about/disease/symptoms.htm). Influenza virus types A and B primarily infect

the nasopharyngeal and oropharyngeal cavities. Influenza A viruses are further categorized into subtypes based on two major surface proteins, hemagglutinin (HA) and neuraminidase (NA). Similarly, Influenza B viruses can also be separated into two major lineages. The infective potential of influenza is frequently underestimated and can result in high morbidity and mortality rates, especially in elderly persons and in high risk patients, such as the very young and immuno-compromised. Typical influenza viral infections in humans have a relatively short incubation period of 1 to 2 days, with symptoms that last about a week including an abrupt onset of fever, sore throat, cough, headache, myalgia, and malaise. When a subject is infected with a highly virulent strain of influenza, these symptoms can progress rapidly to pneumonia and in some circumstances death. Pandemic outbreaks of highly virulent influenza present a serious risk to human and animal health worldwide.

Influenza viruses may be detected and characterized from clinical specimens by several methods that vary in sensitivity, speed, and capability with regard to distinguishing influenza types and subtypes. The CDC Human Influenza Virus Real-Time RT-PCR Diagnostic Panel consists of nucleic acid amplification assays that detect influenza A and B viruses and further characterizes influenza A subtypes A/H1, A/H1pdm09, A/H3, and A/H5 (Asian lineage) and influenza B lineages B/Victoria and B/Yamagata. Components of the CDC Human Influenza Virus Real-Time RT-PCR Diagnostic Panel can be obtained in several kit configurations.

CDC Human Influenza Real-Time RT-PCR Diagnostic Panel FluIVD03		
**Kit Catalog #**	**Contents**	**Application**
FluIVD03-1<br>A/B Typing Kit	**Primer and Probe Sets:** InfA, InfB, RP<br><br>**Controls:** Pooled Influenza Positive Control (PIPC), Human Specimen Control (HSC)	• Detects all influenza A and B viruses
FluIVD03-2<br>A Subtyping Kit	**Primer and Probe Sets:** InfA, H1, H3, pdmInfA, pdmH1, RP<br><br>**Controls:** PIPC	• Specifically detects and differentiates seasonal influenza subtypes; H1, H3, H1pdm09<br>• Detects classical swine influenza viruses and swine triple reassortant viruses[1]
FluIVD03-3<br>A/H5 Subtyping Kit	**Primer and Probe Sets:** InfA, H5a, H5b, RP<br><br>**Controls:** H5VC, HSC	• Specifically detects influenza A/H5 Asian lineage viruses
FluIVD03-4<br>B Lineage Genotyping Kit	**Primer and Probe Sets:** InfB, VIC, YAM, RP<br><br>**Controls:** Influenza B Positive Control (IBPC)	• Specifically detects and differentiates influenza B lineage genotypes; B/Victoria, B/Yamagata

[1]Classical and triple reassortant swine viruses are not seasonal viruses and must be referred to CDC for further confirmation

Laboratories may use the influenza A, B, A/H1, A/H1pdm09, and A/H3 and A/H5 (Asian Lineage) primer and probe sets simultaneously to type and/or subtype suspected influenza positive clinical specimens. Additionally, influenza B viruses may be further characterized as B/Victoria or B/Yamagata using the VIC and YAM primer and probe sets. The CDC Human Influenza Virus Real- Time RT-PCR Diagnostic Panel assists in routine surveillance of seasonal influenza following an algorithm as follows:

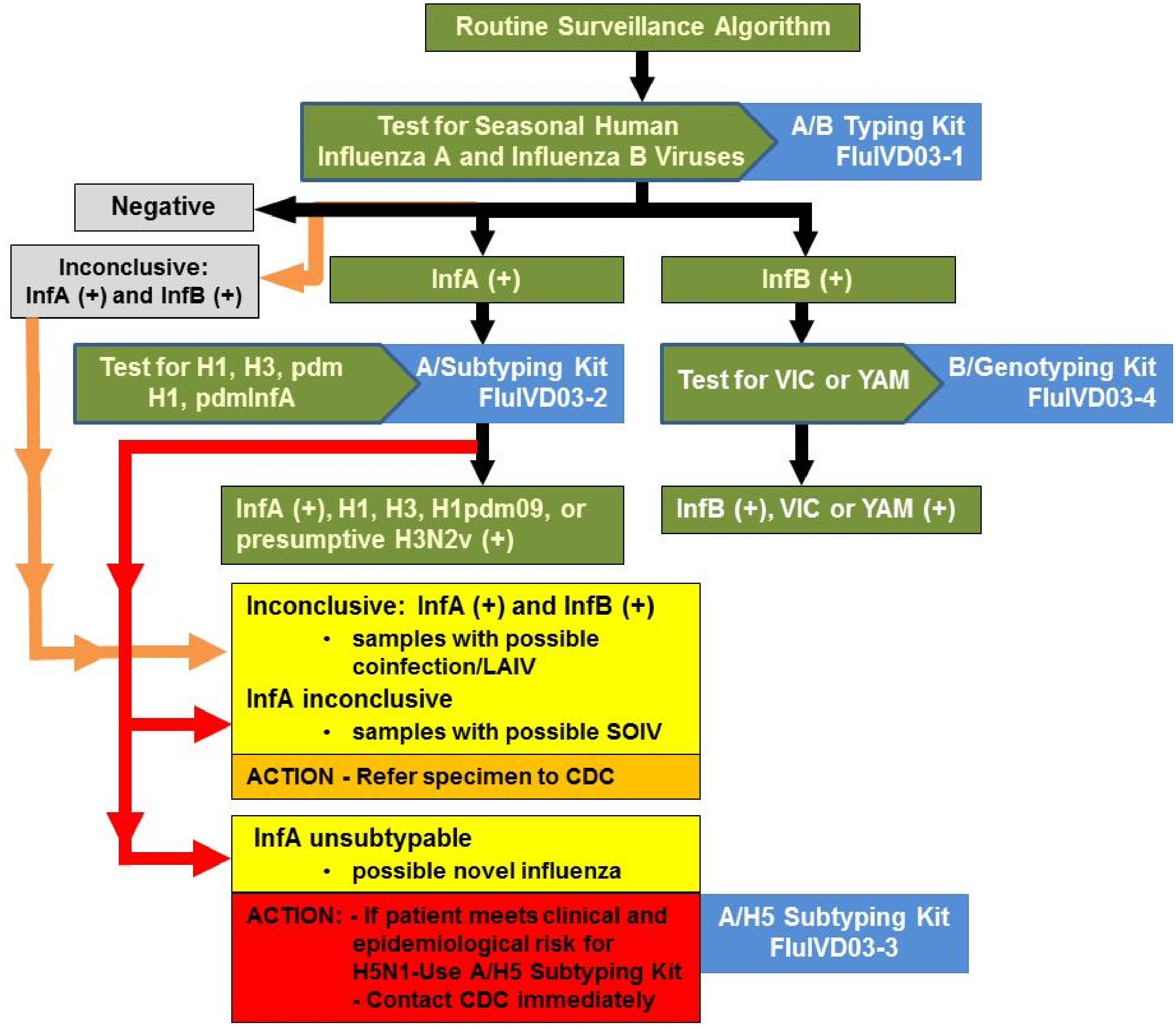

Principles of the Procedure

The Influenza A/B Typing Kit contains components of the CDC Human Influenza Virus Real-Time RT-PCR Diagnostic Panel that is used in rRT-PCR assays on the ABI 7500 Fast Dx Real-Time PCR Instrument. The primer and probe sets contained in the Influenza A/B Typing Kit are designed for the detection of influenza type A and B viruses that infect humans.

The Influenza A/B Typing Kit consists of oligonucleotide primers and dual-labeled hydrolysis (TaqMan®) probes and controls which may be used in rRT-PCR assays for the in vitro qualitative detection and characterization of the human influenza virus RNA in respiratory specimens from patients presenting with influenza-like illness (ILI). The oligonucleotide primers and probes for detection of Influenza A and B viruses were selected from highly conserved regions of the matrix (M) and non-structural (NS) genes, respectively. Detection of viral RNA not only aids in the diagnosis of illness caused by seasonal, newly emerging, and novel influenza viruses in patients with ILI, but also provides epidemiological and surveillance information on influenza and aids in the presumptive laboratory identification of specific novel influenza A viruses.

Summary of Influenza A/B Typing Kit Process

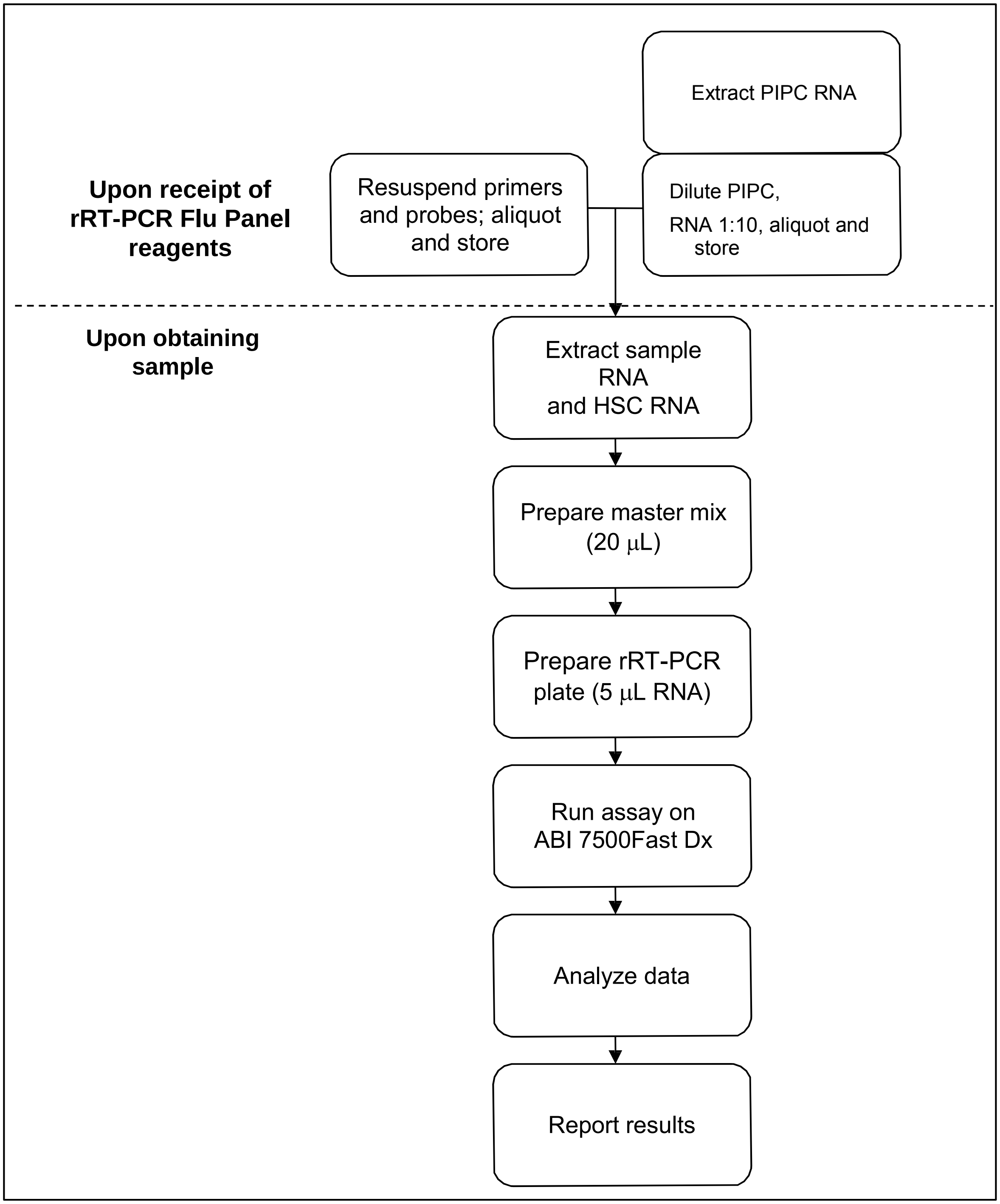

Materials Required (Provided)

Influenza A/B Typing Kit Contents: Catalog # FluIVD03-1
Box #1: Primers and Probes

Reagent Label	*Part #*	*Description*	*Quantity / Tube*	*Reactions / Tube*
InfA-F	SO3304	Influenza A Forward Primer	20 nmol	1000
InfA-R	SO3284	Influenza A Reverse Primer	20 nmol	1000
InfA-P	SO3285	Influenza A Probe (Fam)	5 nmol	1000
InfB-F	SO3311	Influenza B Forward Primer	20 nmol	1000
InfB-R	SO3299	Influenza B Reverse Primer	20 nmol	1000
InfB-P	SO3300	Influenza B Probe (Fam)	5 nmol	1000
RP-F	SO3313	Human RNase P Forward Primer	20 nmol	1000
RP-R	SO3314	Human Rnase P Reverse Primer	20 nmol	1000
RP-P	SO3315	Human Rnase P Probe (Fam)	5 nmol	1000

Box #2: Pooled Influenza Positive Control and Human Specimen Extraction Control

Reagent Label	*Part #*	*Description*	*Quantity / Tube*	*Notes*
PIPC	MR-088	Pooled Influenza Positive Control (PIPC) For use as a positive control with the CDC Human Influenza Virus Real-Time RT-PCR Diagnostic Panel procedure to ensure the detection of seasonal influenza virus A/H1, A/H3, A/H1pdm09 and influenza type B. The PIPC contains noninfectious positive control materials supplied as a liquid, 500 µL per vial, suspended in 0.01 M phosphate buffer saline (PBS) at pH 7.2-7.4. PIPC consists of four (4) different beta-propiolactone treated influenza viruses (influenza A/H1, A/H3, A/H1pdm09, and influenza B) and cultured human cells (A549). PIPC will yield a positive result with the following primer and probe sets: InfA, InfB, H1, H3, pdmInfA, pdmH1, and RP.	1 tube x 500 µL / tube	One thousand 5 µL reactions per tube
Human Specimen Control (HSC)	HS0096	Human Specimen Control (HSC):<br>For use as an RNA extraction procedural control to demonstrate successful recovery of RNA as well as extraction reagent integrity. Purified RNA from the HSC material should yield a positive result with the RP primer and probe set and negative results with all influenza specific markers. The HSC consists of noninfectious (beta propiolactone treated) cultured human cell material supplied as a liquid suspended in 0.01 M PBS at pH 7.2-7.4.	17 tubes x 500 µL / tube	Five 100 µL extractions per tube

Materials Required (But Not Provided)

rRT-PCR Enzyme Mastermix Options

Reagent	Quantity	Catalog No.
Invitrogen SuperScript™ III Platinum® One-Step Quantitative RT-PCR System (without Rox)	100 reactions	11732-020
	500 reactions	11732-088
Invitrogen SuperScript™ III Platinum® One-Step Quantitative RT-PCR System (with Rox)	100 reactions	11745-100
	500 reactions	11745-500
Quanta BioSciences qScript™ One-Step qRT-PCR Kit, Low ROX	50 reactions	95059-050
Quanta BioSciences qScript™ One-Step qRT-PCR Kit, Low ROX	200 reactions	95059-200

RNA Extraction Options

Instrument/Manufacturer	Extraction Kit	Catalog No.
Roche MagNA Pure LC 2.0	Total Nucleic Acid Kit	192 extractions: 03 038 505 001
Roche MagNA Pure Compact	Nucleic Acid Isolation Kit I	32 extractions: 03 730 964 001
Roche MagNA Pure Compact	RNA Isolation Kit	32 extractions: 04 802 993 001
QIAGEN	DSP Viral RNA Mini Kit	50 extractions: 61904
QIAGEN QIAcube	DSP Viral RNA Mini Kit	50 extractions: 61904

Instrument/Manufacturer	Extraction Kit	Catalog No.
bioMérieux NucliSENS® easyMAG® (Automated magnetic extraction reagents sold separately)		EasyMAG® Magnetic Silica (280133)<br><br>EasyMAG® Disposables (280135)<br><br>EasyMAG® Lysis Buffer (280134)<br><br>EasyMAG® Lysis Buffer, 2 mL (200292)<br><br>EasyMAG® Wash Buffers 1,2, and 3 (280130, 280131, 280132)<br><br>Biohit Pipette Tips (280146)

CDC Approved Ancillary Reagents with the CDC Human Influenza Virus Real-Time RT-PCR Diagnostic Panel

Specific lots for the ancillary reagents that are not manufactured under Quality System Regulations (Invitrogen SuperScript™ and Quanta BioSciences qScript™) will be qualified for use with the CDC Human Influenza Virus Real-Time RT-PCR Diagnostic Panel by the CDC Influenza Division. Any lots not specifically qualified by the CDC Influenza Division for use with the CDC Human Influenza Virus Real-Time RT-PCR Diagnostic Panel are not valid for use with this device, and may affect
device performance.

A supplemental cumulative list of the qualified ancillary reagents lots for use with the Flu rRT-PCR Dx Flu Panel is available and can be requested by sending an email to FluSupport@cdc.gov

Any issues related to assay performance or test failure that are suspected to involve ancillary reagents should be reported to the CDC Influenza Division by emailing FluSupport@cdc.gov

Equipment and Consumables Required (But Not Provided)

- Plasticware and consumables
- RNase/DNase-free 1.5 mL polypropylene microcentrifuge tubes
- 100% ethanol (EtOH)
- Disposable gloves
- Molecular grade water (RNase/DNase Free)
- -70°C and -20°C freezer(s)

- 4°C refrigerator
- 96-well cold block
- ABI 7500 Fast Dx Real-Time PCR Instrument (Applied Biosystems, Foster City, CA)
- ABI 7500 Fast Sequence Detection Consumables (Applied Biosystems, Foster City, CA).
    - ABI MicroAmp™ Fast 8-tube strip 0.1 mL, cat #4358293, or ABI MicroAmp™ Fast Optical 96-Well Reaction Plate with Barcode, 0.1 mL, part #4346906, #4346907, or part #4366932 (alternate to 8-strip tubes)
    - ABI MicroAmp™ Optical 8-cap strip, cat #4323032 (required, do not use film)
- Micropipettors (range between 1-10 µL, 10-200 µL and 100-1000 µL)
- Benchtop microcentrifuge

Warnings and Precautions

- For *in-vitro* diagnostic use (IVD).
- Follow standard precautions. All patient specimens and positive controls should be considered potentially infectious and handled accordingly.
- Do not eat, drink, smoke, apply cosmetics or handle contact lenses in areas where reagents and human specimens are handled.
- Handle all specimens as if infectious using safe laboratory procedures. Refer to Biosafety in Microbiological and Biomedical Laboratories (BMBL) 5th Edition BMBL (http://www.cdc.gov/biosafety/) for standard biological safety guidelines for all procedures.
- Specimen processing should be performed in accordance with national biological safety regulations.
- If infection with a novel influenza A virus is suspected based on current clinical and epidemiological screening criteria recommended by public health authorities, specimens should be collected with appropriate infection control precautions for novel virulent influenza viruses and sent to state health departments for testing **IMMEDIATELY**. Virus culture should not be attempted in these cases unless a BSL 3+ facility is available to receive and culture specimens. **Note**: **Novel influenza A viruses are new or re-emergent human strains of influenza A that cause cases or clusters of human disease, as opposed to those strains commonly circulating in humans that cause seasonal epidemics and to which human populations have residual or limited immunity (either by vaccination or previous infection).**
- Performance characteristics have been determined with human upper respiratory specimens (including NPS, NS, TS, NA, NW, DPS/TS) and lower respiratory tract specimens (such as BAL, BW, TA, sputum and lung tissue) from human patients with signs and symptoms of respiratory infection and/or from viral culture.
- Perform all manipulations of live virus samples within a Class II (or higher) biological safety cabinet (BSC).
- Use personal protective equipment such as (but not limited to) gloves and lab coats when handling kit reagents while performing this assay and handling materials including samples, reagents, pipettes, and other equipment and reagents.
- Amplification technologies such as PCR are sensitive to accidental introduction of product from previous amplification reactions. Incorrect results could occur if either the clinical specimen or the real-time reagents used in the amplification step become contaminated by accidental introduction of amplification product (amplicon). Workflow in the laboratory should proceed in a unidirectional manner.

- Maintain separate areas for assay setup and handling of nucleic acids.
- Always check the expiration date prior to use. Do not use expired reagent. Do not substitute or mix reagent from different kit lots or from other manufacturers.
- Change aerosol barrier pipette tips between all manual liquid transfers.
- During preparation of samples, compliance with good laboratory techniques is essential to minimize the risk of cross-contamination between samples, and the inadvertent introduction of nucleases into samples during and after the extraction procedure. Proper aseptic technique should always be used when working with nucleic acids.
- Maintain separate, dedicated equipment (e.g., pipettes, microcentrifuges) and supplies (e.g., microcentrifuge tubes, pipette tips) for assay setup and handling of extracted nucleic acids.
- Wear a clean lab coat and powder-free disposable gloves (not previously worn) when setting up assays.
- Change gloves between samples and whenever contamination is suspected.
- Keep reagent and reaction tubes capped or covered as much as possible.
- Primers, probes (including aliquots), and enzyme master mix must be thawed and maintained on cold block at all times during preparation and use.
- Work surfaces, pipettes, and centrifuges should be cleaned and decontaminated with cleaning products such as 5% bleach, “DNAzap™” or “RNase AWAY®” to minimize risk of nucleic acid contamination. Residual bleach should be removed using 70% ethanol.

- Reagents, master mix, and RNA should be maintained on cold block or on ice during preparation and use to ensure stability.
- Dispose of unused kit reagents and human specimens according to local, state, and federal regulations.

Reagent Storage, Handling, and Stability

- Store all primers and probes at 2-8°C until re-hydrated for use; Store all control materials (HSC, PIPC) at ≤ -20°C.
- Always check the expiration date prior to use. Do not use expired reagents.
- Protect fluorogenic probes from light.
- Primers, probes (including aliquots), and enzyme master mix must be thawed and kept on cold block at all times during preparation and use.
- Do not refreeze probes.
- Controls and aliquots of controls must be thawed and kept on ice at all times during preparation and use.

Specimen Collection, Handling, and Storage

Inadequate or inappropriate specimen collection, storage, and transport are likely to yield false negative test results. Training in specimen collection is highly recommended due to the importance of specimen quality. CLSI MM13-P may be referenced as an appropriate resource.

- Collecting the Specimen
  - Follow specimen collection devices manufacturer instructions for proper collection methods.
  - Swab specimens should be collected using only swabs with a synthetic tip, such as nylon or Dacron®, and an aluminum or plastic shaft. Calcium alginate swabs are unacceptable and cotton swabs with wooden shafts are not recommended.
- Transporting Specimens
  - Ensure that, when transporting human respiratory specimens, all applicable regulations for the transport of etiologic agents are met. Human respiratory specimens, to be tested within 72 hours post-collection, should be transported refrigerated at 2–8°C. Alternatively, specimens may be frozen and transported for testing.
  - Respiratory specimens should be collected and placed into viral transport media (VTM) as described by CDC and WHO guidelines.
  - For more information refer to WHO “Guidelines for the collection of clinical specimens during field investigation of outbreaks”, http://www.who.int/csr/resources/publications/surveillance/whocdscsredc2004.pdf, and/or www.cdc.gov/h1n1flu/specimencollection.htm.
- Storing Specimens
  - Specimens received cold should be stored refrigerated (2–8°C) for up to 72 hours before processing. Store any residual specimens at ≤ -70°C.
  - Although optimal performance is met when testing fresh specimens within 72 hours of collection, performance has been demonstrated with frozen specimens. If testing of a fresh specimen is not possible within 72 hours storage at 2–8°C, the specimen may be frozen at ≤ -70°C and tested at a later time.
  - Specimens received frozen should be stored at ≤ -70°C until processing. Store any residual specimens at ≤ -70°C.
- Storing Purified Nucleic Acid
  - Store purified nucleic acids at ≤ -70°C.

Specimen Referral to CDC

- Referring a specimen to the CDC
  - Ship all specimens and related RNA overnight to CDC.
  - Ship frozen specimens on dry ice and non-frozen specimens on cold packs. Ship extracted RNA on dry ice.
  - Refer to the International Air Transport Association (IATA - www.iata.org) for requirements for shipment of human or potentially infectious biological specimens.
  - Prior to shipping, notify CDC Influenza Division (see contact information below) that you are sending specimens.
  - Fill out the Influenza Specimen Submission Form completely – include specimen type and Ct values detected by your laboratory.

- Send all samples to the following recipient:

Virology, Surveillance and Diagnosis Branch, Influenza Division
Centers for Disease Control and Prevention
c/o STAT, MS G-16
Attention: Dr. Stephen Lindstrom
1600 Clifton Rd., Atlanta, GA 30333
Phone: (404) 639-1587 or (404) 639-3591
Fax: (404) 639-0080

The emergency contact number for CDC Emergency Operations Center (EOC) is 770-488-7100.

Please see the following WHO reference for more details:
http://www.who.int/csr/resources/publications/surveillance/CDS_EPR_ARO_2006_1.pdf

Reagent and Controls Preparation

Primer and Probe Preparation:

1. Upon receipt, store lyophilized primers and probes at 2-8°C.
2. Rehydration
   a. Remove primers and probes from 2-8°C.
   b. Allow primers and probes to sit at ambient temperature for 15 minutes.
   c. Quickly centrifuge dried primers and probes to collected pellet in the bottom of the tube.
   d. Pipette 0.5 ml (500 µL) of 10 mM Tris, pH 7.4-8.2 or PCR-grade water into each dried PCR primer or probe.
   e. Allow primers and probes to fully rehydrate for at least 15 minutes at room temperature.
   f. After primers and probes are fully rehydrated, pulse vortex to ensure a homogenous solution.
3. Prepare a Combined Oligonucleotide Mix for Each Marker Set
   a. For each marker set (e.g. InfA), pipette the entire volume (0.5 mL) of the reconstituted forward and reverse primers and probe into a single new, nuclease-free, sterile tube (henceforth, the primer/probe mix).
   b. Pulse vortex the combined primer/probe mix to ensure a homogenous solution.
4. Aliquot
   a. Label five new, nuclease-free, sterile tubes for each combined primer/probe mix with the following information:
      - Primer or probe name
      - Kit lot #
      - Expiration date
   b. Aliquot 300 µL of each combined primer/probe mix into respective labeled tubes and store at -20°C.

5. Storage
    a. After rehydration
        i. Aliquots of primers and probes are stored at -20°C or below until expiration date as long as QC requirements are met.
        ii. Thawed aliquots of primers and probes may be stored at 2-8°C for up to 3 months. Combined primers/probe mix aliquots should be stored in the dark.

Human Specimen Control (HSC) Preparation:

1. Reagent
    a. Noninfectious cultured human cell material supplied as a liquid suspension in 0.01 M PBS.
    b. Volume: 0.5 mL per vial.
2. Storage
    a. Store at -20°C or below upon receipt. Do not dilute.
3. Procedure
    a. Human Specimen Control must be extracted and processed with each batch of samples to be tested. The final volume of eluted RNA should equal the volume of extracted control material. For example, 100 µL of control material should result in 100 µL of RNA extract.
    b. Do not dilute extracted RNA prior to testing.

Pooled Influenza Positive Control (PIPC) Preparation:

1. Reagent
    a. Inactivated, noninfectious influenza virus preparation supplied as a liquid suspension in 0.01 M PBS.
    b. Control contains four different influenza viruses representing influenza A/H1, A/H3, A/H1pdm09, and influenza B viruses in cultured human cells.
    c. Volume: 0.5 mL yields approximately 5 mL of positive control RNA.
2. Storage
    a. Store at -20°C or below upon receipt. Do not dilute.
3. Procedure
    a. PIPC must be extracted prior to use. The final volume of eluted RNA should equal the volume of extracted control material. For example, 100 µL of control material should result in 100 µL of RNA extract (1:1).
    b. Dilute the RNA 1:10 with nuclease free water.
    c. Label one new nuclease-free, sterile, tube for each single-use aliquot with the following information:
        - Control RNA name and 1:10 dilution
        - Kit lot #
        - Expiration date
    d. Dispense the 1:10 diluted RNA into single-use aliquots and store at -20°C or below for up to 6 months. Recommended aliquot volume (but not required):
        - 20 µL aliquots for use with the Influenza A/B Typing Kit screening assays.
    e. Use one aliquot per run. Discard after use. Do not use residual RNA.

General Preparation

Equipment Preparation

Clean and decontaminate all work surfaces, pipettes, centrifuges, and other equipment prior to use. Decontamination agents should be used including 5% bleach, 70% ethanol, and ***DNAzap***™ or ***RNase AWAY***® to minimize the risk of nucleic acid contamination.

Reagent Preparation

NOTE: All reagents should be kept on ice or cold rack during assay preparation.

Primers and Probes Reagents

- Thaw frozen aliquots of primer/probe mix. *Thawed aliquots may be stored at 2-8°C in the dark for up to 3 months.* ***Do not re-freeze****.*
- Vortex each combined primer/probe mix for 15 seconds.
- Briefly centrifuge each primer/probe mix.
- Place combined primer/probe mix in cold rack during master mix preparation.

Real-time RT-PCR Reagents **(Important: Select Appropriate Enzyme System)**

Invitrogen SuperScript™ III Platinum® One-Step Quantitative RT-PCR System

- Place Invitrogen 2X PCR Master Mix and Superscript III RT/Platinum Taq enzyme mix in a cold rack at 2-8°C.
- Completely thaw the 2X PCR Master Mix vial.
- Mix the 2X PCR Master Mix by inversion 10 times.
- Briefly centrifuge 2X PCR Master Mix and Superscript III RT/Platinum Taq enzyme mix then place in cold rack.

OR

Quanta BioSciences qScript™ One-Step qRT-PCR Kit, Low ROX

- Place Quanta qScript™ One-Step Master Mix (2X) and qScript™ One-Step Reverse Transcriptase in a cold rack at 2-8°C.
- Completely thaw the One-Step Master Mix (2X) vial.
- Mix the One-Step Master Mix (2X) by inversion 10 times.
- Briefly centrifuge One-Step Master Mix (2X) and qScript™ One-Step Reverse Transcriptase then place in cold rack.

Nucleic Acid extraction

Performance of the CDC Human Influenza Virus Real-Time RT-PCR Diagnostic Panel is dependent upon the amount and quality of template RNA purified from human specimens. The following commercially available RNA extraction kits and procedures have been qualified and validated for recovery and purity of RNA for use with the panel:

Qiagen QIAamp® DSP Viral RNA Mini Kit

Recommendation(s): Utilize 100 µL of sample and elute with 100 µL of buffer, or utilize 140 µL of sample and elute with 140 µL of buffer.

<u>Qiagen QiaCube with the QIAamp DSP Viral RNA Mini Kit</u>
Kit: Qiagen QIAamp® DSP Viral RNA Mini Kit
Recommendations: Utilize 140 µL of sample and elute with 100 µL of buffer.

<u>Roche MagNA Pure LC</u>
Kit: Roche MagNA Pure Total Nucleic Acid Kit
Protocol: Total NA External_lysis
Recommendation(s): Utilize 100 µL of sample and 300 µL of lysis buffer (total sample volume for input into LC is 400 µL). Elution volume is 100 µL. These volumes should be available on the LC software.

<u>Roche MagNA Pure Compact</u>
Kit: Roche MagNA Pure Nucleic Acid Isolation Kit I
Protocol: Total_NA_Plasma100_400
Recommendation(s): Utilize 100 µL of sample and 300 µL of lysis buffer (total sample volume for input into Compact is 400 µL). Elution volume is 100 µL.

<u>Roche MagNA Pure Compact</u>
Kit: Roche MagNA Pure RNA Isolation Kit
Protocol: RNA_Tissue Protocol
Recommendation(s): Utilize 100 µL of sample and 250 µL of lysis buffer (total sample volume for input into Compact is 350 µL). Elution volume is 100 µL.

<u>bioMérieux NucliSENS® easyMAG® Instrument</u>
Protocol: General protocol (not for blood) using "Off-board Lysis" reagent settings
Recommendation(s): Utilize 100 µL of sample and 1000 µL of lysis buffer (total sample volume for input into Compact is 1100 µL). Incubate for 10 minutes at room temperature. Elution volume is 100 µL.

<u>Manufacturer's recommended procedures are to be used for sample extraction.</u>
Disclaimer: Names of vendors or manufacturers are provided as examples of suitable product sources. Inclusion does not imply endorsement by the Centers for Disease Control and Prevention.

Assay Setup

IMPORTANT INFORMATION!
The CDC Human Influenza Virus Real-Time RT-PCR Diagnostic Panel consists of individual assays that can be utilized to meet laboratory testing algorithms, for example to perform high throughput screening using the Influenza A/B Typing Assay. However, CDC strongly recommends that laboratories perform subtype characterization of all the influenza A positives with the Influenza A Subtyping Assay. Current recommendations can be found on the FluSupport SharePoint site at https://partner.cdc.gov/Sites/NCIRD/FS/default.aspx.

Master Mix Preparation / Plate Setup

1. In the assay preparation area, label a sterile, nuclease-free, 1.5 mL tube for each reaction master mix to be prepared for each specific master mix / marker to be prepared (i.e. InfA, InfB, etc.).
2. Determine the number of reactions (N) to be prepared per assay.
3. Calculate the amount of each reagent to be added to the tube for each master mix by multiplying the number of reactions (samples plus controls) per marker by the volume of reagent indicated in **Figure 1**.

NOTE: It is necessary to make excess reaction master mix to allow for pipetting error.

Example: If number of samples (n) including controls = 1 to 14, then N = n + 1

If number of samples (n) including controls > 15, then N = n + 2

Figure 1. Steps and Calculations for Master Mix Preparation (Important: Select Appropriate Enzyme System)

Invitrogen SuperScript™ III Platinum® One-Step Quantitative RT-PCR System

Step #	Reagent	Vol. of Reagent Added per Reaction
1	Nuclease-free Water	N x 5.5 µL
2	Combined Primer/Probe Mix	N x 1.5 µL
3	SuperScript™ III RT/Platinum® *Taq* Mix	N x 0.5 µL
4	2X PCR Master Mix	N x 12.5 µL
	**Total Volume**	**N x 20.0 µL**

OR

Quanta BioSciences qScript™ One-Step qRT-PCR Kit, Low ROX

Step #	Reagent	Vol. of Reagent Added per Reaction
1	Nuclease-free Water	N x 5.5 µL
2	Combined Primer/Probe Mix	N x 1.5 µL
3	Quanta qScript™ One-Step Reverse Transcriptase	N x 0.5 µL
4	One-Step Master Mix (2X)	N x 12.5 µL
	**Total Volume**	**N x 20.0 µL**

4. In a sterile labeled 1.5 mL tube, prepare master mix for each marker set to be tested by first calculating the amount of each reagent to be added for each primer/probe set reaction master mix required for the test being performed.
5. In the assay preparation area, dispense reagents into each respective labeled 1.5 mL microcentrifuge tube. After addition of the water, mix reaction mixtures by pipetting up and down. ***Do not vortex***.
6. Centrifuge for 5 seconds to collect contents at the bottom of the tube, and then place the tube in a cold rack.
7. Set up reaction strip tubes or plates in a 96-well cooler rack.
8. Dispense 20 µL of each master mix into the appropriate wells going across the row as shown below (**Figure 2**):

Figure 2. Influenza A/B Typing Assay: Example of Reaction Master Mix Plate Set-up

	1	2	3	4	5	6	7	8	9	10	11	12
**A**	InfA	InfA	InfA	InfA	InfA	InfA	InfA	InfA	InfA	InfA	InfA	InfA
**B**	InfB	InfB	InfB	InfB	InfB	InfB	InfB	InfB	InfB	InfB	InfB	InfB
**C**	RP	RP	RP	RP	RP	RP	RP	RP	RP	RP	RP	RP
**D**	empty	empty	empty	empty	empty	empty	empty	empty	empty	empty	empty	empty
**E**	empty	InfA	InfA	InfA	InfA	InfA	InfA	InfA	InfA	InfA	InfA	empty
**F**	empty	InfB	InfB	InfB	InfB	InfB	InfB	InfB	InfB	InfB	InfB	empty
**G**	empty	RP	RP	RP	RP	RP	RP	RP	RP	RP	RP	empty
**H**	empty	empty	empty	empty	empty	empty	empty	empty	empty	empty	empty	empty

9. Prior to moving to the nucleic acid handling area, prepare the NTC reactions for column #1 in the assay preparation area.
10. Pipette 5 µL of nuclease-free water into the NTC sample wells. Securely cap NTC wells before proceeding.
11. Cover the entire reaction plate and move the reaction plate to the specimen nucleic acid handling area.

Template Addition

1. Gently vortex nucleic acid sample tubes for approximately 5 seconds.
2. After centrifugation, place extracted nucleic acid sample tubes in the cold rack.
3. Samples should be added to column 2-11 (column 1, 11 (bottom half), and 12 are for controls) to the specific assay that is being tested as illustrated in **Figure 3**. Carefully pipette 5.0 µL of the first sample into all the wells labeled for that sample (i.e. Sample "S1" down column #2). *Keep other sample wells covered during addition. Change tips after each addition.*
4. Securely cap the column to which the sample has been added to prevent cross contamination and to ensure sample tracking.
5. Change gloves often and when necessary to avoid contamination.
6. Repeat steps #3 and #4 for the remaining samples.
7. Add 5 µL of Human Specimen Control (HSC) extracted sample to the HSC wells (**Figure 3**, column 11). Securely cap wells after addition. This is applicable to any plate set-up.
8. Cover the entire reaction plate and move the reaction plate to the positive template control handling area.

Assay Control Addition

1. Pipette 5 µL of PIPC RNA to the sample wells of column 12(**Figure 3).** Securely cap wells after addition of the control RNA.

   NOTE: *<u>If using 8-tube strips</u>, label the TAB of each strip to indicate sample position.* ***DO NOT LABEL THE TOPS OF THE REACTION TUBES!***
2. Briefly centrifuge reaction tube strips for 10-15 seconds. After centrifugation return to cold rack.

   NOTE*: <u>If using 96-well plates</u>, centrifuge plates for 30 seconds at 500 x g, 4°C.*

Figure 3. Influenza A/B Typing Kit: Example of Sample and Control Set-up

	1	2	3	4	5	6	7	8	9	10	11	12
**A**	NTC	S1	S3	S5	S7	S9	S11	S13	S15	S17	S19	PIPC
**B**	NTC	S1	S3	S5	S7	S9	S11	S13	S15	S17	S19	PIPC
**C**	NTC	S1	S3	S5	S7	S9	S11	S13	S15	S17	S19	PIPC
**D**	empty	empty	empty	empty	empty	empty	empty	empty	empty	empty	empty	empty
**E**	empty	S2	S4	S6	S8	S10	S12	S14	S16	S18	HSC	empty
**F**	empty	S2	S4	S6	S8	S10	S12	S14	S16	S18	HSC	empty
**G**	empty	S2	S4	S6	S8	S10	S12	S14	S16	S18	HSC	empty
**H**	empty	empty	empty	empty	empty	empty	empty	empty	empty	empty	empty	empty

Create a Run Template on the ABI 7500 Fast Dx Real-time PCR Instrument

(Required if no template exists)

If the template already exists on your instrument please proceed to the **<u>RUNNING A TEST</u>** section.

1. Launch the ABI 7500 Fast Dx Real-time PCR System by double clicking on the ABI 7500 Fast Dx System icon on the desktop.
2. A new window should appear, select **Create New Document** from the menu.

Figure 4. New Document Wizard Window

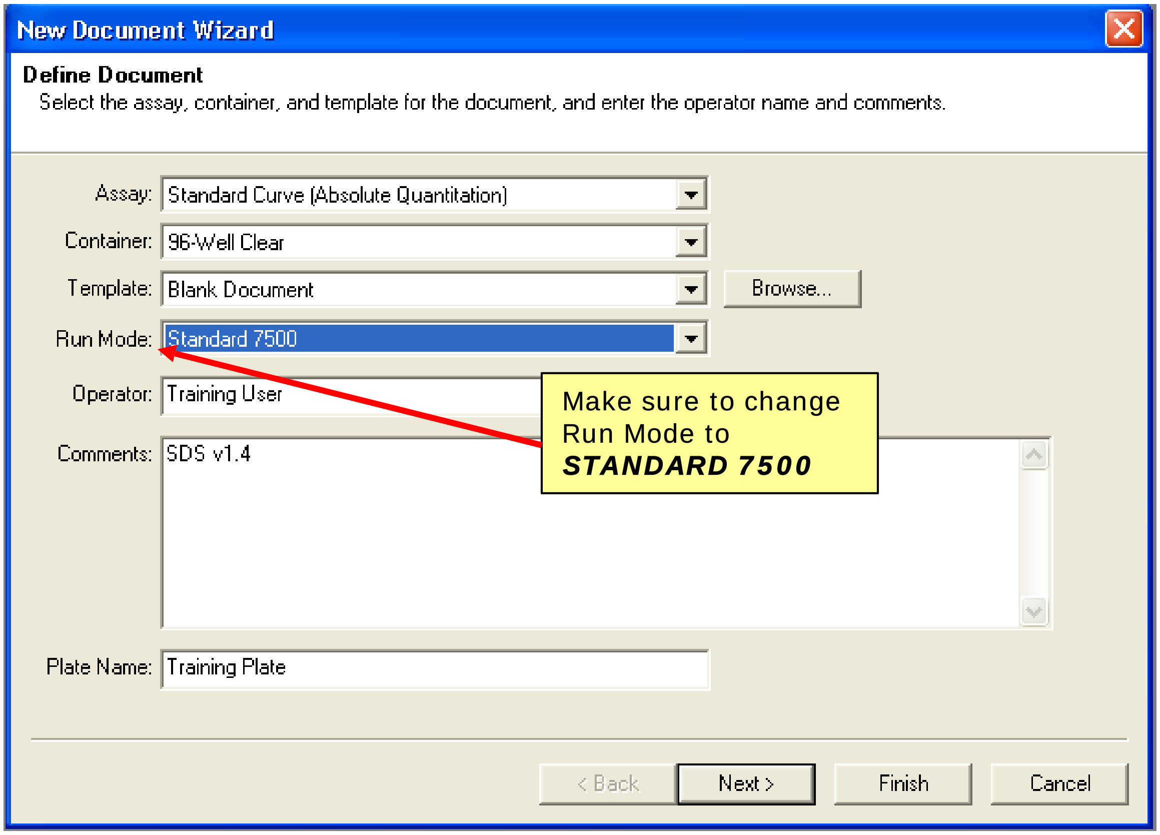

3. The **New Document Wizard** screen in **Figure 4** will appear. Select:
   a. Assay: **Standard Curve (Absolute Quantitation)**
   b. Container: **96-Well Clear**
   c. Template: **Blank Document**
   d. Run Mode: **Standard 7500**
   e. Operator: ***Your Name***
   f. Comments: **SDS v1.4**
   g. Plate Name: ***Your Choice***
4. After making selections click **Next** at the bottom of the window.

Figure 5. Creating New Detectors

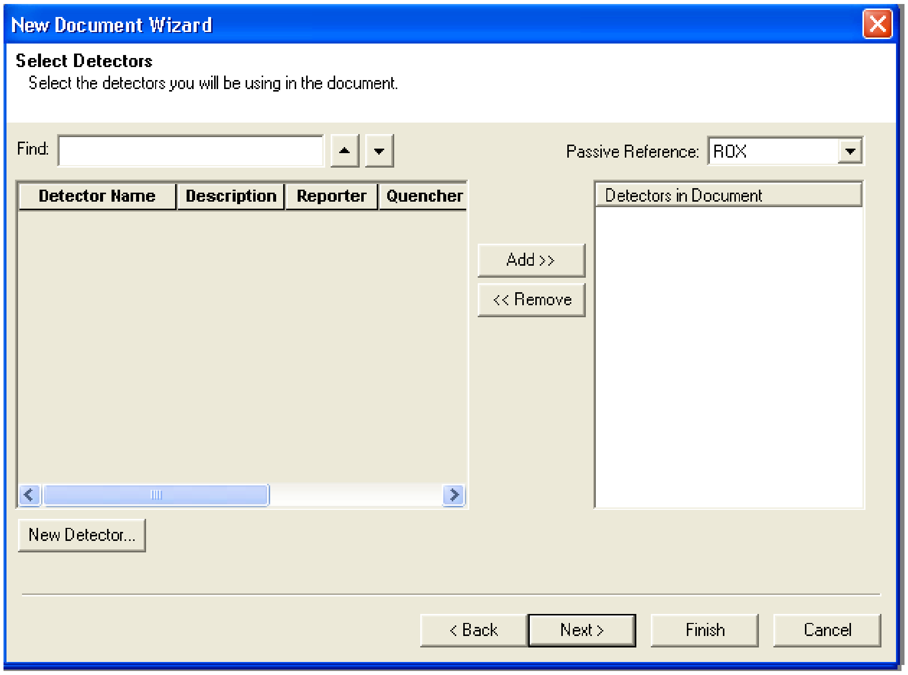

5. After selecting next, the ***Select Detectors*** screen (**Figure 5**) will appear.
6. Click the **New Detector** button (see **Figure 5**).
7. The **New Detector** window will appear (**Figure 6**). A new detector will need to be defined for each influenza primer and probe set. Creating these detectors will enable you to analyze each primer and probe set individually at the end of the reaction.

Figure 6. New Detector Window

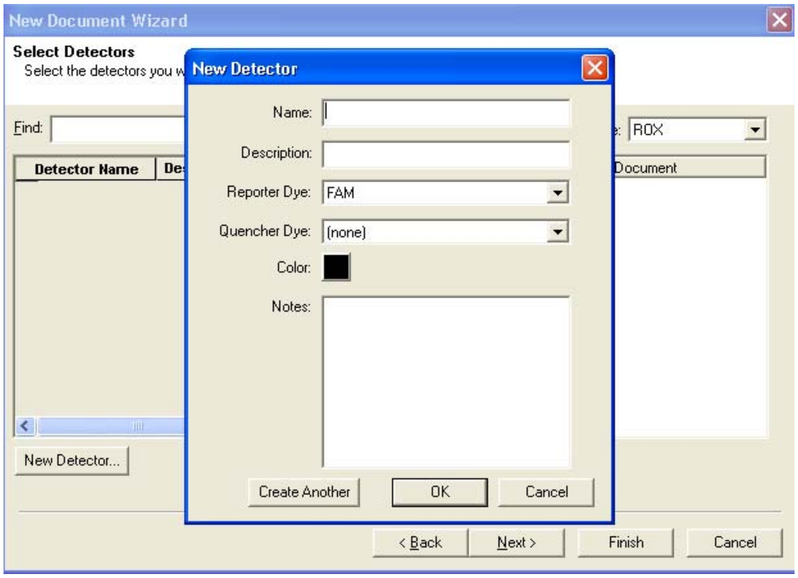

8. Start by creating the InfA Detector. Include the following:
   a. Name:**InfA**
   b. Description: *leave blank*c.
   c. Reporter Dye: **FAM**
   d. Quencher Dye: **(none)**
   e. Color: *to change the color of the detector indicator do the following:*
      ⇒ Click on the color square to reveal the color chart
      ⇒ Select orange as the color by clicking on the orange square
      ⇒ After selecting color click **OK** to return to the New Detector screen
   f. Click the **OK** button of the New Detector screen to return to the screen shown in **Figure 5**.
9. Repeat step 6-8 for each influenza target in the A/B Typing Kit.

Name	Reporter Dye	Quencher Dye
InfA	FAM	(none)
InfB	FAM	(none)
RP	FAM	(none)

10. After each Detector is added, the **Detector Name**, **Description**, **Reporter** and **Quencher** fields will become populated in the **Select Detectors** screen (**Figure 7**).
11. Before proceeding, the newly created detectors must be added to the document. To add the new detectors to the document, click **ADD** (see **Figure 7**). Detector names will appear on the right hand side of the **Select Detectors** window (**Figure 7**).

Figure 7. Adding New Detectors to Document

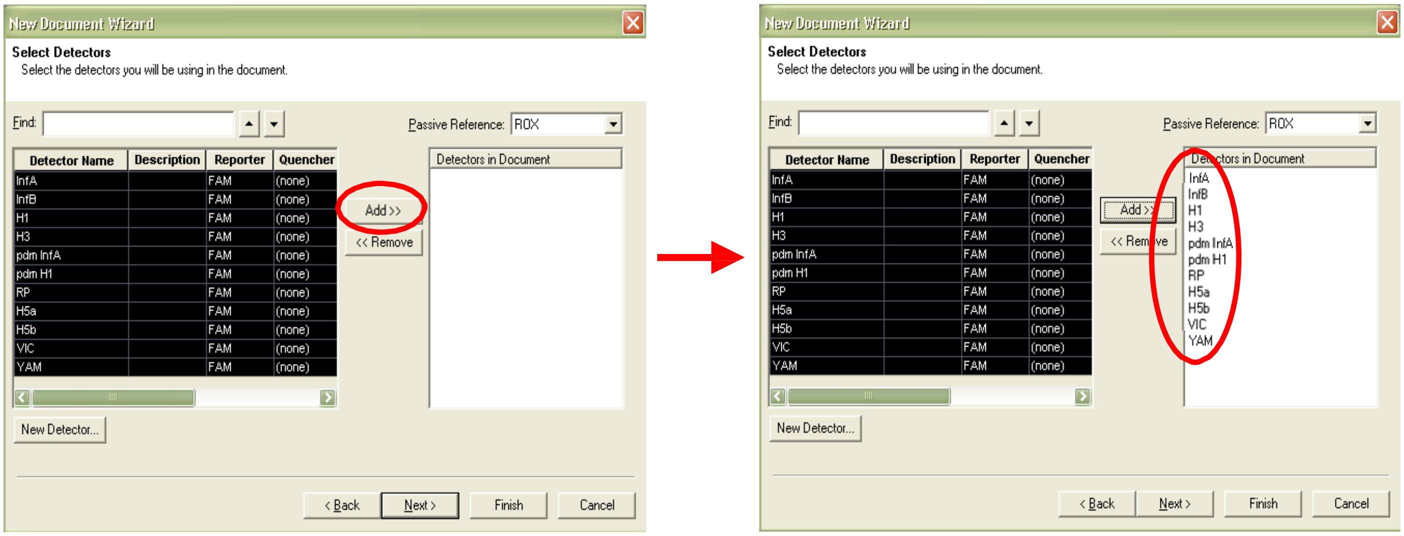

12. Once all detectors have been added, select **(none)** for **Passive Reference** at the top right hand drop down menu (**Figure 8**).

Figure 8. Select Passive Reference

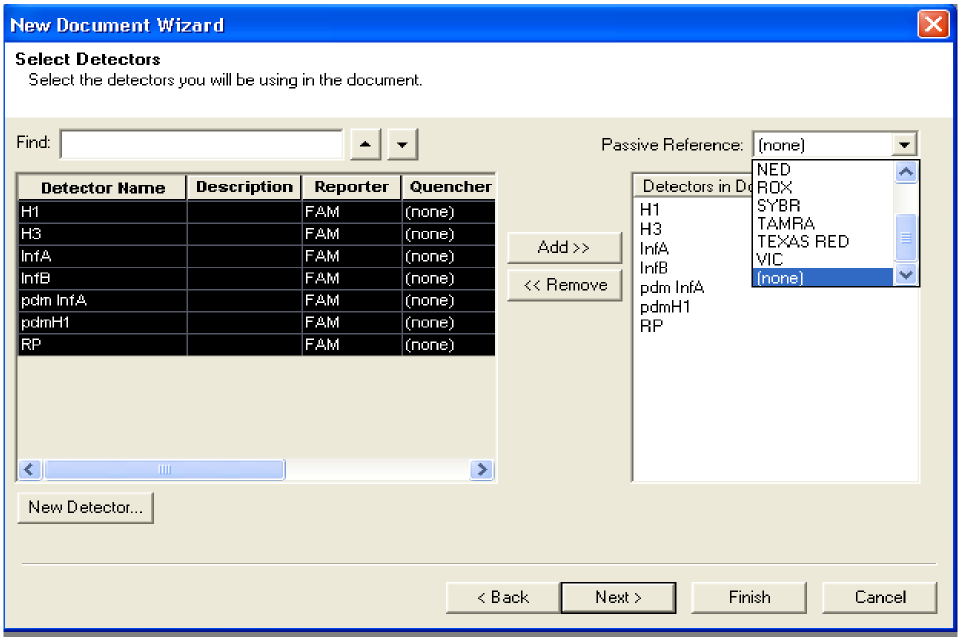

Passive reference should be set to "(none)" as described above.

13. Click **Next** at the bottom of the **Select Detectors** window to proceed to the **Set Up Sample Plate** window (**Figure 8**).
14. In the **Set Up Sample Plate** window (**Figure 9**), use your mouse to select row A from the lower portion of the window, in the spreadsheet (see **Figure 9**).
15. In the top portion of the window, select detector **InfA**. A check will appear next to the detector you have selected (**Figure 9**). You will also notice the row in the spreadsheet will be populated with a colored "U" icon to indicate which detector you've selected.
16. Repeat step 14-15 for each detector that will be used in the assay.

Figure 9. Sample Plate Set-up

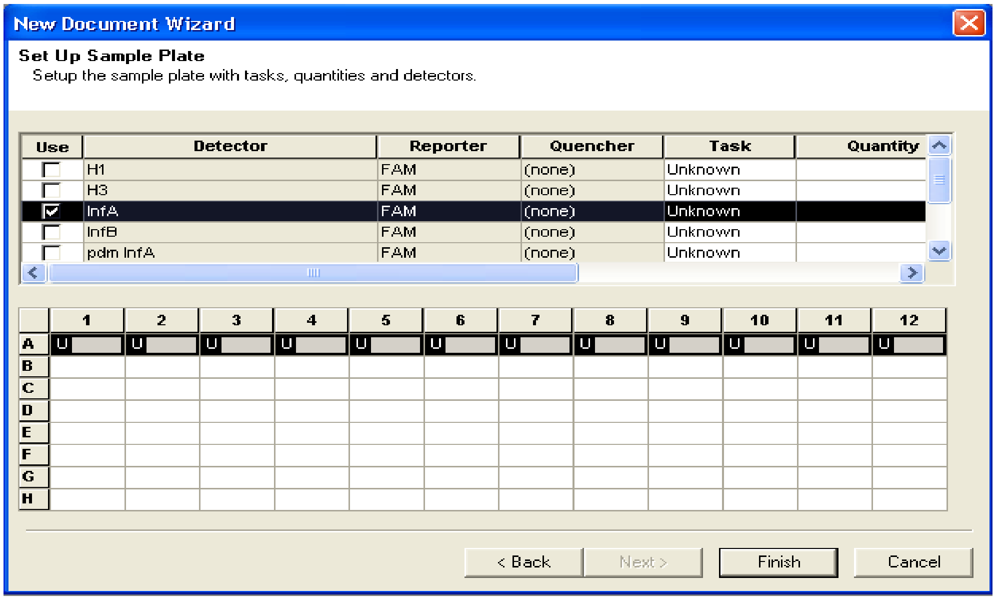

17. Select **Finish** after detectors have been assigned to their respective rows. (**Figure 10**).

Figure 10. Finished Sample Plate Set-up

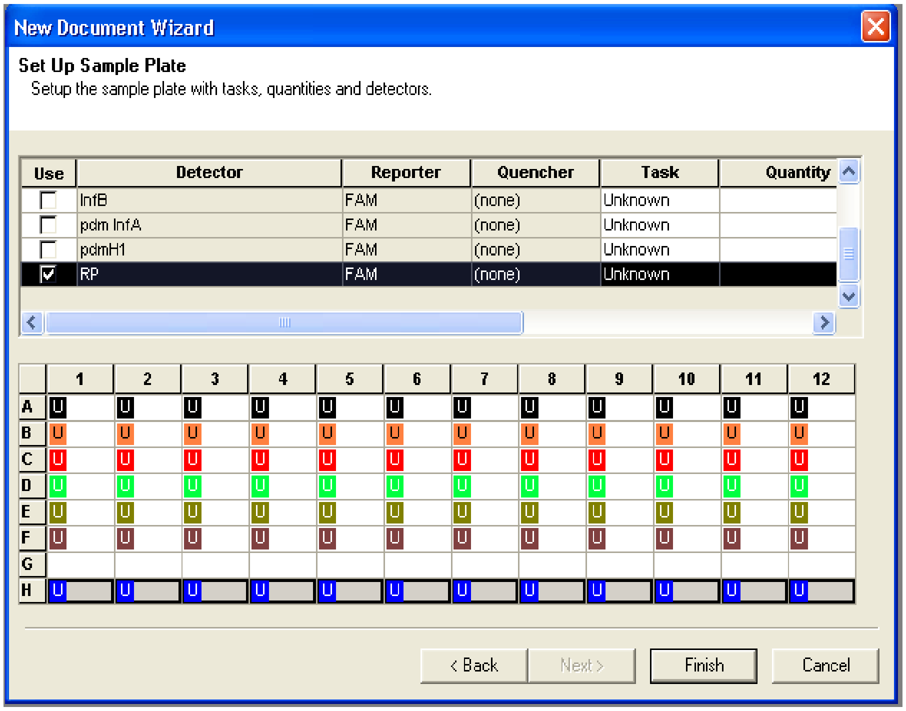

18. After clicking "Finish", there will be a brief pause allowing the ABI 7500 Fast Dx to initialize. This initialization is followed by a clicking noise. ***Note: The machine must be turned on for initialization.***
19. After initialization, the **Plate** tab of the Setup (**Figure 11)** will appear.

20. Each well of the plate should contain colored U icons that correspond with the detector labels that were previously chosen. To confirm detector assignments, select **Tools** from the file menu, then select **Detector Manager.**

Figure 11. Plate Set-up Window

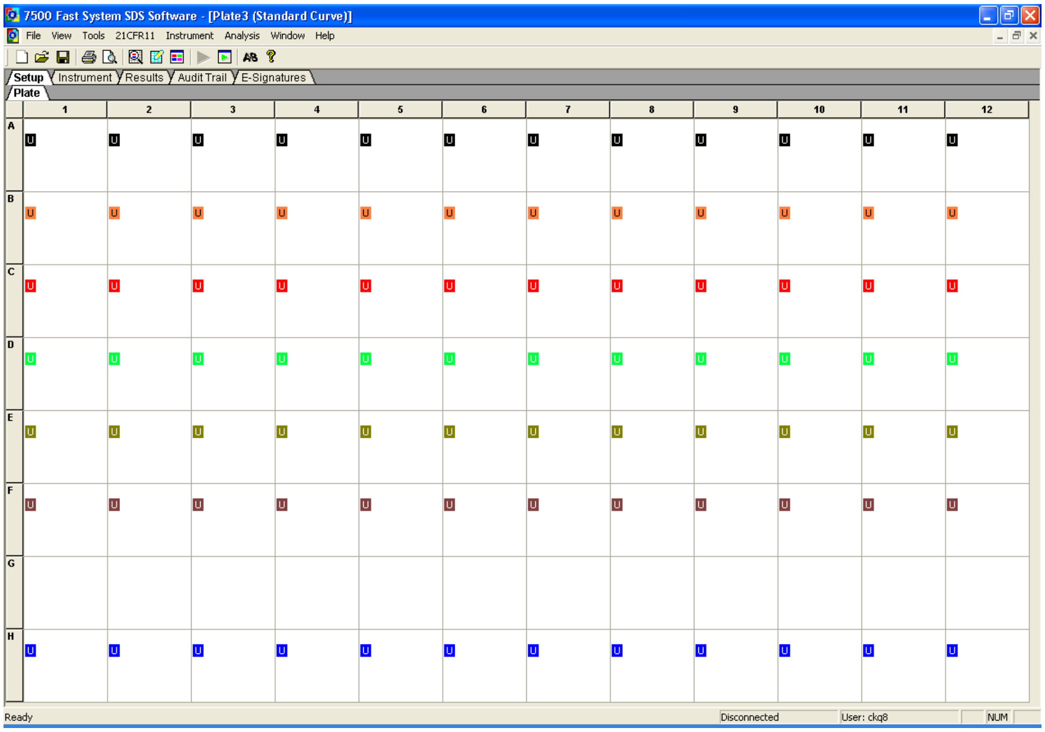

21. The Detector Manager window will appear (**Figure 12**).

Figure 12. Detector Manager Window

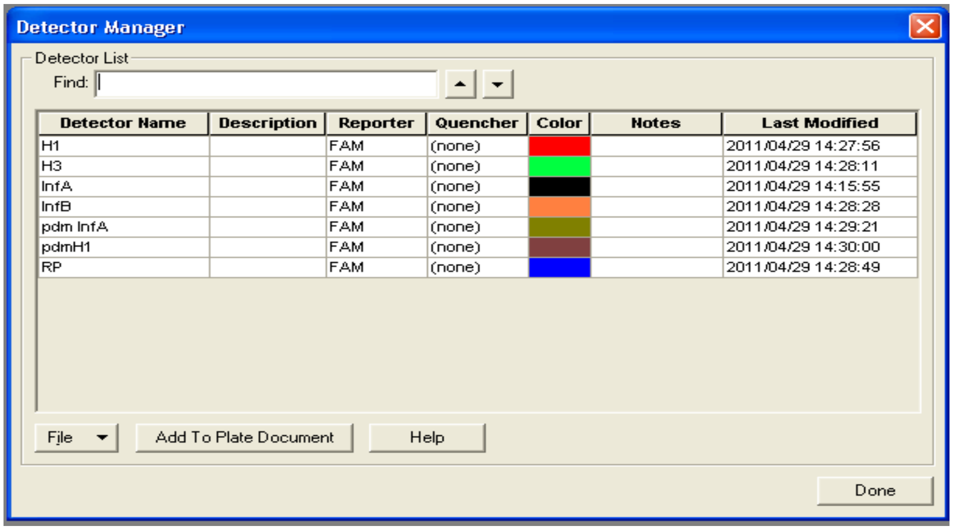

22. Confirm all influenza detectors are included and that each influenza target has a **Reporter** set to **FAM** and the **Quencher** is set to **(none)**.
23. If all detectors are present, select **Done**. The detector information has been created and assigned to wells on the plate.

Defining the Instrument Settings

(Important: Use Appropriate Settings for Invitrogen SuperScript™ III or Quanta qScript™ Enzyme)

1. After detectors have been created and assigned, proceed to instrument set up.
2. Select the **Instrument** tab to define thermal cycling conditions.
3. Modify the thermal cycling conditions as follows (**Figure 13**):

Invitrogen SuperScript™ III Platinum® One-Step Quantitative RT-PCR System

a. In Stage 1, Set to **30 min** at **50°C**; **1 Rep**.
b. In Stage 2, Set to **2.0 min** at **95°C**; **1 Rep**.
c. In Stage 3, Step 1 set to **15 sec** at **95°C**.
d. In Stage 3, Step 2 set to **30 sec** at **55.0°C.**
e. In Stage 3, Reps should be set to **45.**
f. Under **Settings** (**Figure 13**), bottom left-hand box, change volume to 25 µL.
g. Under **Settings**, **Run Mode** selection should be **Standard 7500**.
h. Step 2 of Stage 3 should be highlighted in yellow to indicate data collection (see **Figure 13**).

OR

Quanta BioSciences qScript™ One-Step qRT-PCR Kit, Low ROX

a. In Stage 1, Set to **30 min** at **50°C**; **1 Rep**.
b. In Stage 2, Set to **5.0 min** at **95°C**; **1 Rep**.
c. In Stage 3, Step 1 set to **15 sec** at **95°C**.
d. In Stage 3, Step 2 set to **30 sec** at **55.0°C**.
e. In Stage 3, Reps should be set to **45**.
f. Under **Settings (Figure 13)**, bottom left-hand box, change volume to 25 µL.
g. Under **Settings**, **Run Mode** selection should be **Standard 7500**.
h. Step 2 of Stage 3 should be highlighted in yellow to indicate data collection (**see Figure 13**).

Figure 13. Instrument Window

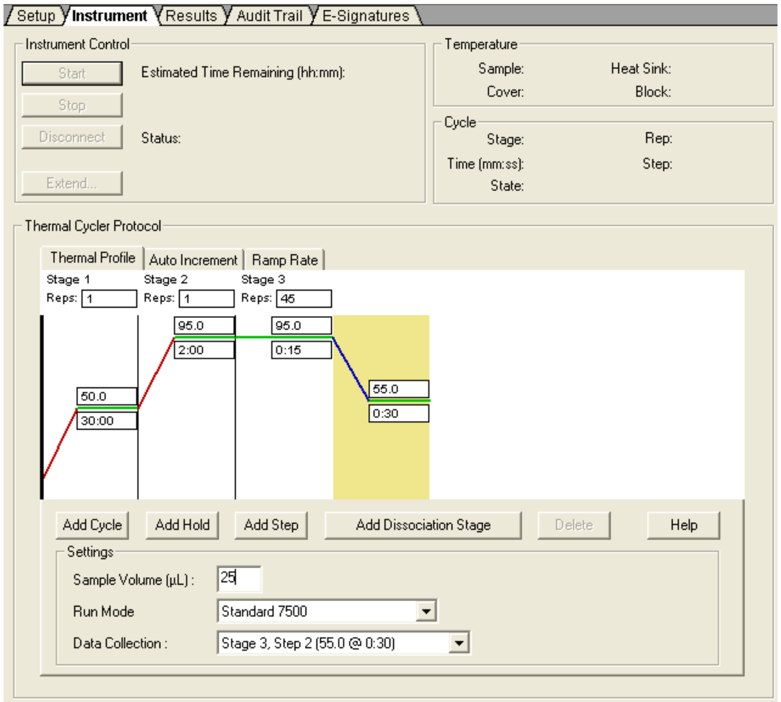

4. After making changes to the **Instrument** tab, the template file is ready to be saved. To save the template, select **File** from the top menu, then select **Save As**. Since the two enzyme options have different instrument settings it is recommended that the template be saved with a name indicating the enzyme option.
5. Save the template as **Influenza AB Typing Kit SuperScript** or **Influenza AB Typing Kit qScript** as appropriate in the desktop folder labeled "***ABI Run Templates***" (*you must create this folder*). Save as type should be SDS Templates (*.sdt) (**Figure 14**).

Figure 14. Saving Template

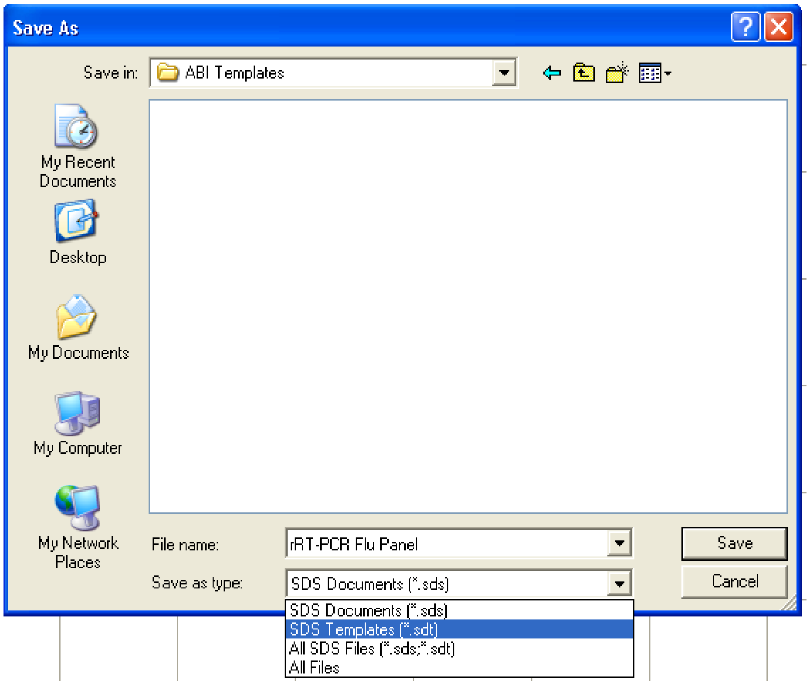

Running a Test

1. Turn on the ABI 7500 Fast Dx Real-Time PCR Instrument.
2. Launch the ABI 7500 Fast Dx Real-time PCR System by double clicking on the 7500 Fast Dx System icon on the desktop.
3. A new window should appear, select **Open Existing Document** from the menu.
4. Navigate to select your ABI Run Template folder from the desktop.
5. Double click on the appropriate template file (**Influenza AB Typing Kit SuperScript** or **Influenza AB Typing Kit qScript**).
6. There will be a brief pause allowing the ABI 7500 Fast Dx Real-Time PCR Instrument to initialize. This initialization is followed by a clicking noise. ***Note: The machine must be turned on for initialization.***

Figure 15. Plate Set-up Window

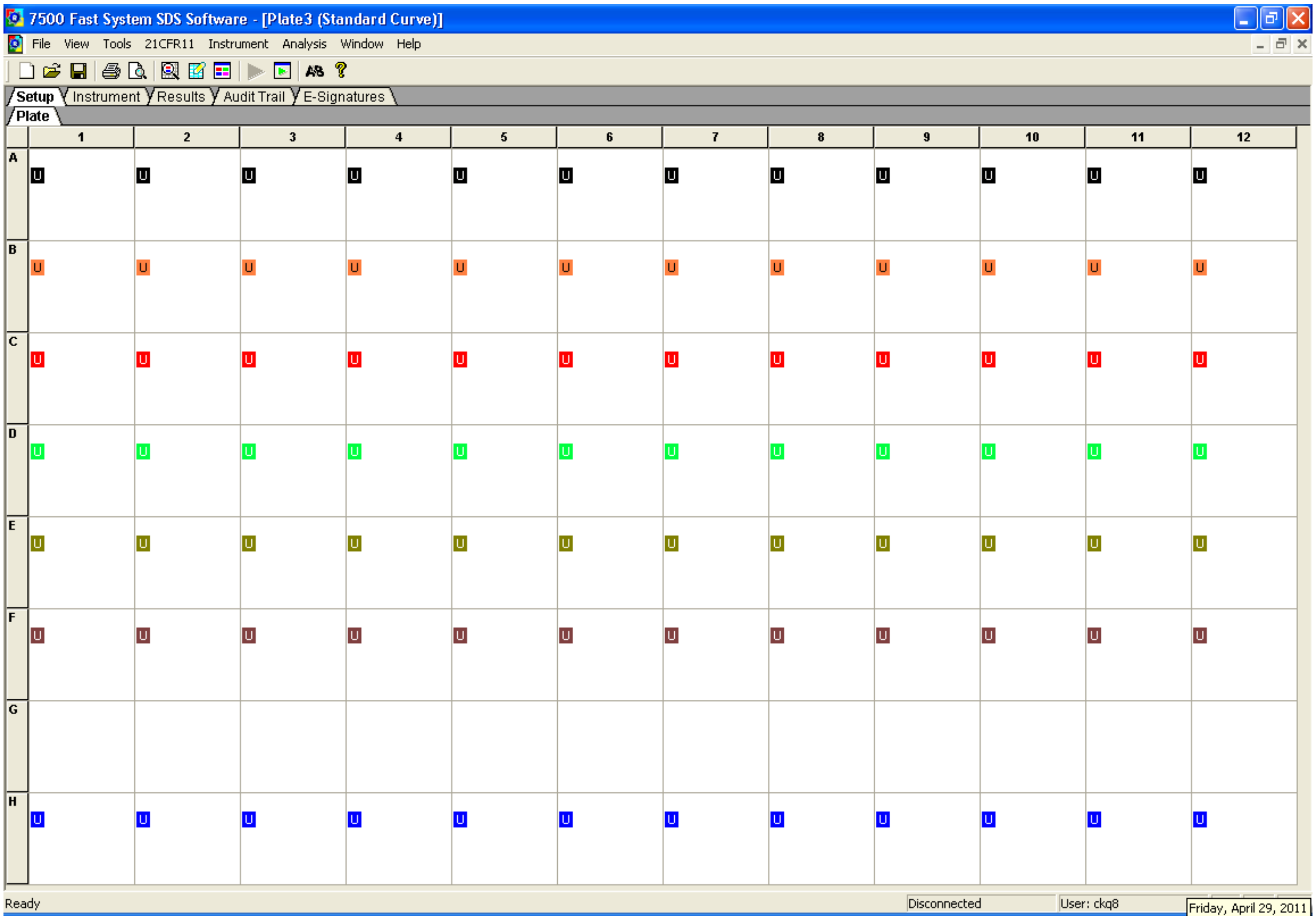

7. After the instrument initializes, a plate map will appear (**Figure 15**). The detectors and controls should already be labeled as they were assigned in the original template.

8. Click the **Well Inspector** icon 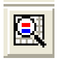 from the top menu.
9. Highlight specimen wells of interest on the plate map.
10. Type sample identifiers to **Sample Name** box in the **Well Inspector** window (**Figure 16**).

Figure 16. Labeling Wells

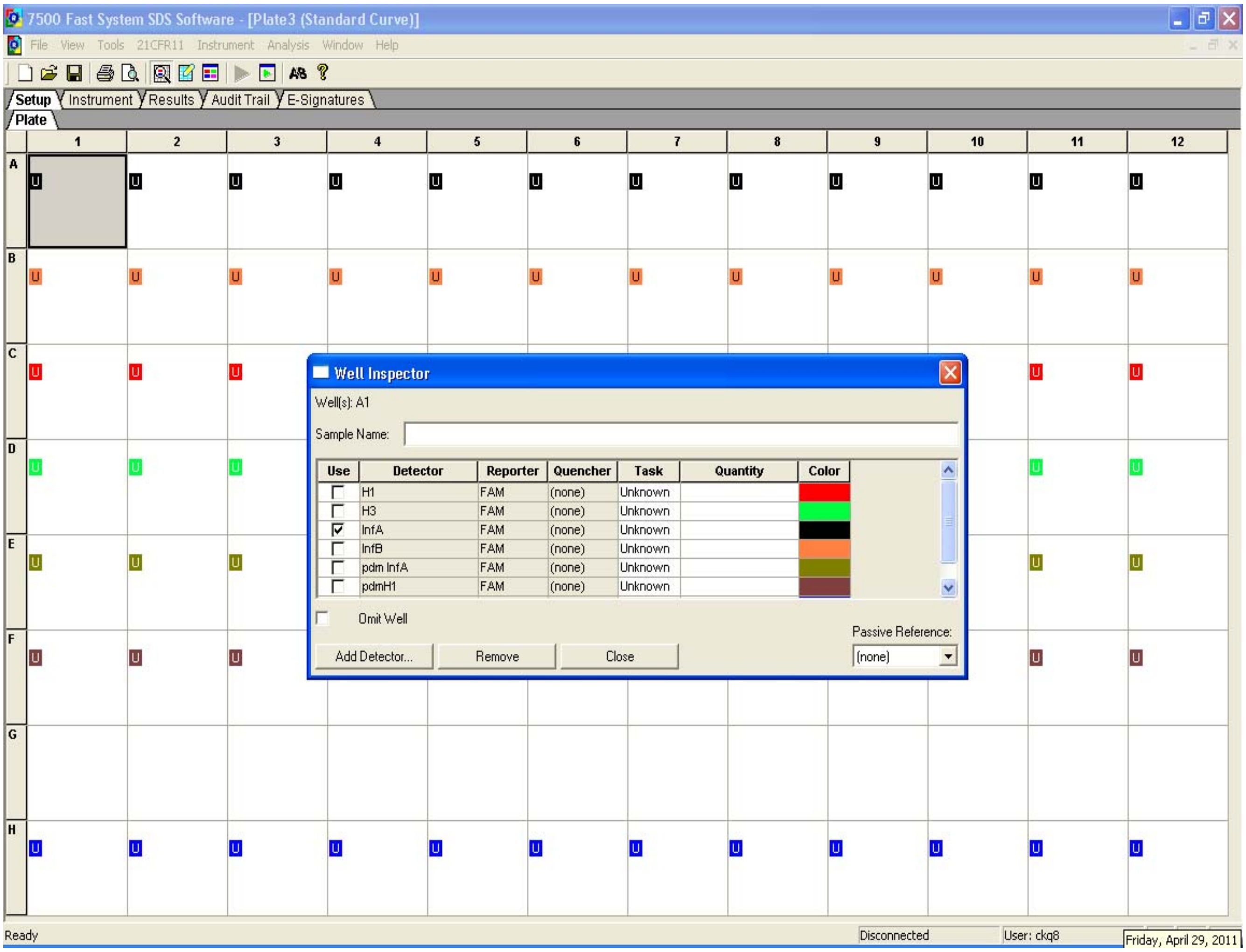

11. Repeat steps 9-10 until all sample identifiers are added to the plate setup.
12. Once all specimen and control identifiers are added click the **Close** button on the **Well Inspector** window to return to the **Plate** set up tab.
13. Click the **Instrument** tab at the upper left corner.
14. The reaction conditions, volumes, and type of 7500 reaction should already be loaded. (**Figure 17**).

Figure 17. Instrument Settings

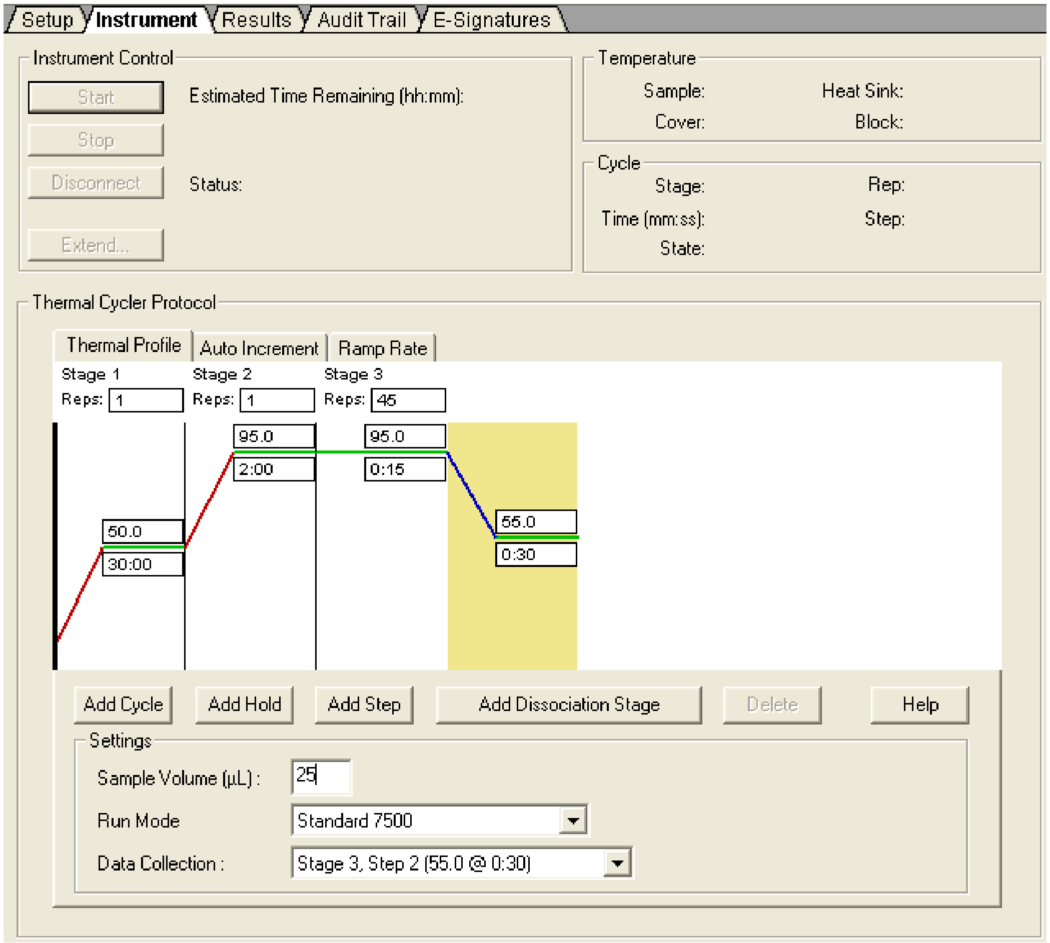

15. Ensure settings are correct (refer to the *Defining Instrument Settings*).
16. Before proceeding, the run file must be saved; from the main menu, select **File,** then **Save As**. Save in appropriate run folder designation.
17. Load the plate into the plate holder in the instrument. Ensure that the plate is properly aligned in the holder.
18. Once the run file is saved, click the **Start** button. *Note: The run should take approximately 1hr and 45 minutes to complete*

Data Analysis

1. After the run has completed, select the **Results** tab at the upper left corner of the software.
2. Select the **Amplification Plot** tab to view the raw data (**Figure 18**).

Figure 18. Amplification Plot Window

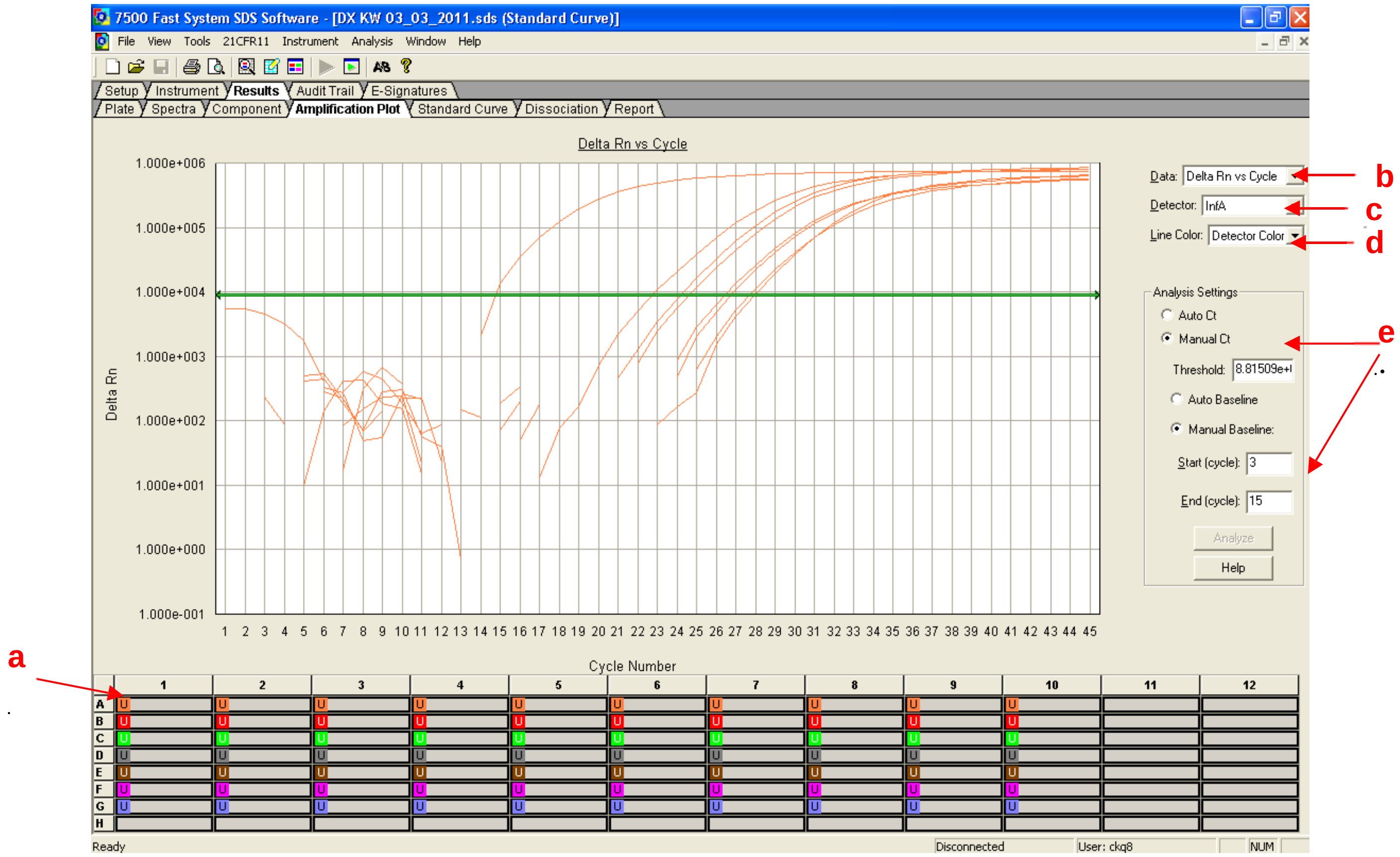

3. Start by highlighting all the samples from the run; to do this, click on the upper left hand box **(a)** of the sample wells (**Figure 18**). All the growth curves should appear on the graph.
4. On the right hand side of the window **(b)**, the **Data** drop down selection should be set to **Delta Rn vs. Cycle**.
5. Select **InfA** from **(c)**, the **Detector** drop down menu, using the downward arrow.
    a. Please note that each detector is analyzed individually to reflect different performance profiles of each primer and probe set.
6. In the **Line Color** drop down **(d)**, **Detector Color** should be selected.
7. Under **Analysis Settings** select **Manual Ct** **(e)**.
    a. Do not change the **Manual Baseline** default numbers.
8. Using the mouse, click and drag the red threshold line until it lies within the exponential phase of the fluorescence curves and above any background signal (**Figure 19**).

Figure 19. Amplification Plot

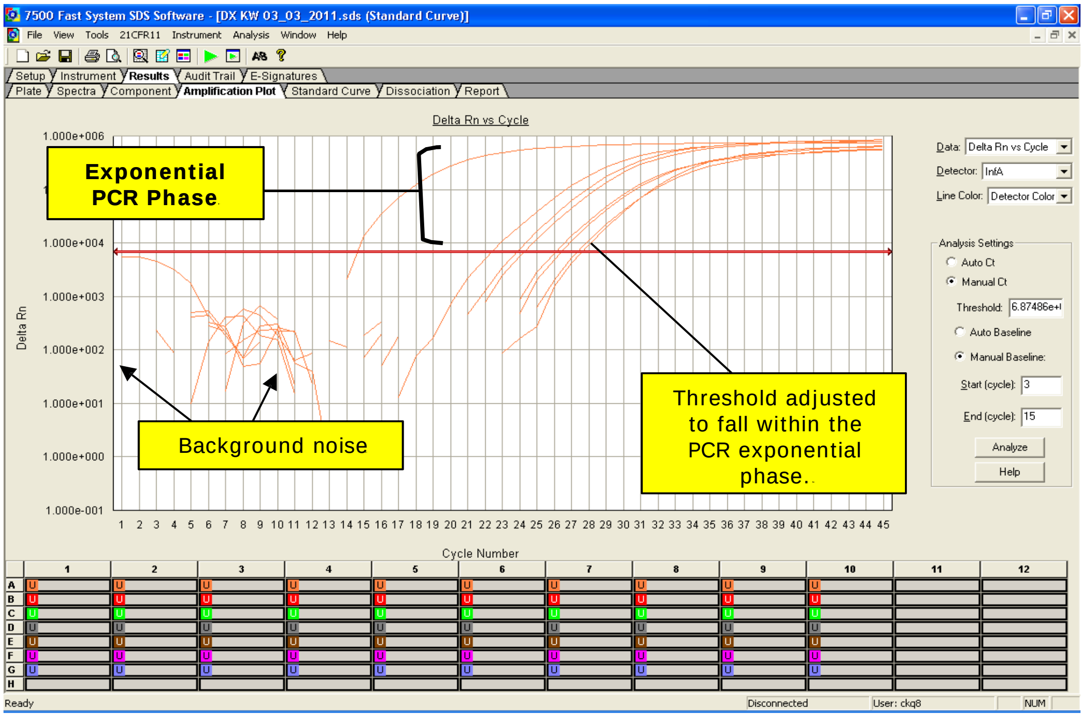

9. Click the **Analyze** button in the lower right corner of the window. The red threshold line will turn to green, indicating the data has been analyzed.
10. Repeat steps 5-9 to analyze results generated for each set of markers (i.e. InfA, InfB, etc).
11. Save analysis file by selecting **File** then **Save As** from the main menu.
12. After completing analysis for each of the markers, select the **Report** tab above the graph to display the Ct values. To filter report by sample name in ascending or descending order, simply click on **Sample Name** in the table.

Figure 20. Report

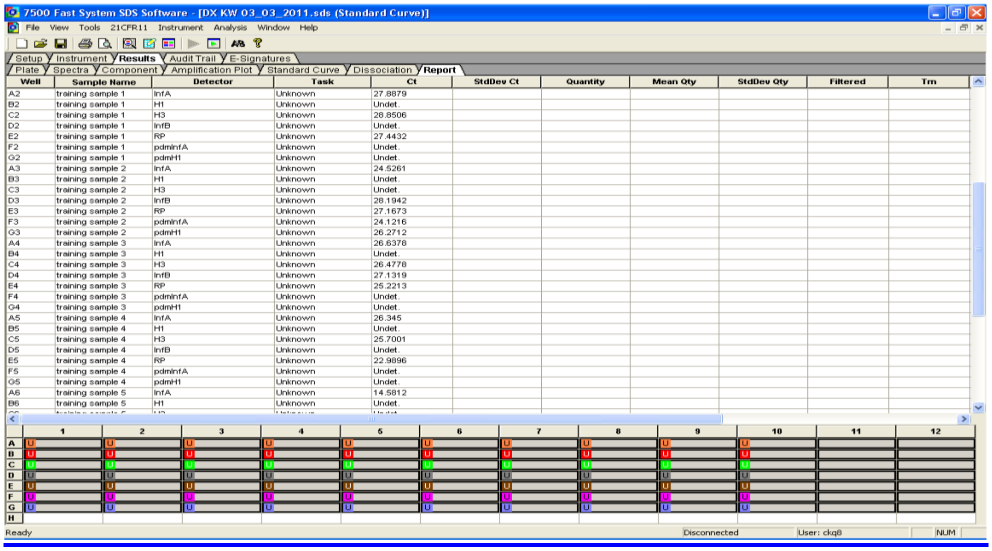

Interpretation of Results and Reporting

Extraction and Positive Control Results and Interpretation

No Template Control (NTC)

- The NTC consists of using nuclease-free water in the rRT-PCR reactions instead of RNA. The NTC reactions for all primer and probe sets should not exhibit fluorescence growth curves that cross the threshold line. If any of the NTC reactions exhibit a growth curve that crosses the cycle threshold, sample contamination may have occurred. Invalidate the run and repeat the assay with strict adherence to the guidelines.

Pooled Influenza Positive Control (PIPC)

- The PIPC consists of four different influenza viruses representing influenza A/H1N1, A/H3N2, A/H1pdm09, and influenza B viruses suspended in cultured human cells (A549). Purified RNA from the PIPC will yield a positive result with the following primer and probe sets: InfA, InfB, H1, H3, pdmInfA, pdmH1, and RP.

Human Specimen Control (HSC) (Extraction Control)

- The HSC control consists of noninfectious cultured human cell (A549) material. The HSC is used as an RNA extraction procedural control to demonstrate successful recovery of RNA as well as extraction reagent integrity. Purified RNA from the HSC should yield a positive result with the RP primer and probe set and negative results with all influenza specific markers.

Expected Performance of Controls Included in the Influenza A/B Typing Assay

Control Type	Internal Control Name	Used to Monitor	InfA	InfB	RP	Expected Ct Values
Positive	PIPC	Substantial reagent failure including primer and probe integrity	+	+	+	< 38.00 Ct
Negative	NTC	Reagent and/or environmental contamination	-	-	-	None detected
Extraction	HSC	Failure in lysis and extraction procedure	-	-	+	< 38.00 Ct

If the controls do not exhibit the expected performance as described, the assay may have been set up and/or executed improperly, or reagent or equipment malfunction could have occurred. Invalidate the run and re-test.

RNase P (Extraction Control)

- All clinical samples should exhibit fluorescence growth curves in the RNase P reaction that cross the threshold line within 38.00 cycles (< 38.00 Ct), thus indicating the presence of the human RNase P gene. Failure to detect RNase P in

any clinical specimens may indicate:

- Improper extraction of nucleic acid from clinical materials resulting in loss of RNA and/or RNA degradation.
- Absence of sufficient human cellular material due to poor collection or loss of specimen integrity.
- Improper assay set up and execution.
- Reagent or equipment malfunction.

➢ If the RP assay does not produce a positive result for human clinical specimens, interpret as follows:

- If the InfB or InfA are positive even in the absence of a positive RP, the influenza result should be considered valid. It is possible, that some samples may fail to exhibit RNase P growth curves due to low cell numbers in the original clinical sample. A negative RP signal does not preclude the presence of influenza virus RNA in a clinical specimen.
- If all influenza markers <u>AND</u> RNase P are negative for the specimen, the result should be considered inconclusive for the specimen. If residual specimen is available, repeat the extraction procedure and repeat the test. If all markers remain negative after re-test,
report the results as inconclusive and a new specimen should be collected if possible.

➢ The RP assay may be negative when testing virus culture samples.

<u>Influenza Markers (InfA, InfB)</u>

➢ When all controls exhibit the expected performance, a specimen is considered negative if all influenza marker (InfA, InfB) cycle threshold growth curves **DO NOT** cross the threshold line within 38.00 cycles (< 38.00 Ct) **AND** the RNase P growth curve **DOES** cross the threshold line within 38.00 cycles (< 38.00 Ct).

➢ When all controls exhibit the expected performance, a specimen is considered positive for influenza if the influenza marker (InfA, InfB) cycle threshold growth curve crosses the threshold line within 38.00 cycles (< 38.00 Ct). The RNase P may or may not be positive as described above, but the influenza result is still valid. When testing tissue culture derived samples, the Rnase P result is likely to yield negative / not detected result due to the absence of the human RNase P target.

➢ When all controls exhibit the expected performance and the growth curves for the influenza markers (InfA, InfB) **AND** the RNase P marker **DO NOT** cross the cycle threshold growth curve within 38.00 cycles (< 38.00 Ct), the result is inconclusive. The extracted RNA from the specimen should be re-tested. If residual RNA is not available, re-extract RNA from residual specimen and re-test. If the re-tested sample is negative for all markers and all controls exhibit the expected performance, the result is “Inconclusive.”

Influenza A/B Typing Assay Results Interpretation Guide

The table below lists the expected results for the Influenza A/B Typing Assay. If results are obtained that do not follow these guidelines, re-extract and re-test the sample. If repeat testing yields similar results, contact CDC Technical Support for consultation and possible specimen referral as some results may indicate novel or emerging influenza viruses. See pages 12 and 55 for referral and contact information.

Influenza A/B Typing Assay

InfA	InfB	RP	Result Interpretation[a]	Report for CDC Surveillance	Notes and Special Guidance
+	-	±	Influenza A Detected; Subtyping not performed	Influenza A	
-	+	±	Influenza B Detected	Influenza B	
-	-	+	Influenza NOT Detected	Not Detected	
+	+	±	Report: Influenza A Detected and Influenza B Detected; Refer to CDC for further characterization.	Inconclusive	If original result, re-extract and re-test. If repeat, report as possible co-infection. Specimen should be referred to CDC for further characterization; possible co-infection or LAIV detection
-	-	-	Inconclusive Failure of controls	Inconclusive	If original result, re-extract and re-test. If repeat, report as Inconclusive. Obtain new specimen if possible.

[a]**Laboratories should report their diagnostic result as appropriate and in compliance with their specific reporting system.**

While this device can be used as a test for influenza A and B only, CDC strongly recommends that laboratories subtype all the influenza A positives with the Influenza A Subtyping Assay. The CDC recommends maintaining the enhanced surveillance efforts by state and local health departments, hospitals, and clinicians to identify patients that may be transmitting a possible novel or newly emerging influenza virus.

Standards-Based Electronic Laboratory Reporting for Influenza

Background

This section contains the recommendations for uniform coding and vocabulary for CDC Human Influenza Virus Real-time RT-PCR Diagnostic Panel.

The following information is provided to assist the performing laboratory in complying with new federal guidelines for the meaningful use of electronic health information systems. The implementation of adopted standards should be harmonized across all performing laboratories to ensure semantic interoperability to better support electronic data exchange.

The CDC developer of this assay through collaboration has established Standard English terminology for the test name and test results with the testing community and expert knowledge of the processes involved. It is recognized that this terminology will differ in countries outside the United States. However, through the use of national and international agreements, it is possible to establish a universal set of codes and terms to accurately characterize laboratory observations. Recommendations in this package insert apply to the reporting of results of this assay only within the United States.

Process for achieving uniformity in laboratory test results

The laboratory performing the influenza assay may utilize a Laboratory Information Management Systems (LIMS) with connections to a hospital or medical system Electronic Health Record (EHR). The coding systems include LOINC - Logical Observation Identifiers Names and Codes (LOINC® -- http://www.loinc.org) and SNOMED CT – Systematic Nomenclature of Medicine--Clinical Terms (http://www.ihtsdo.org/). These coding systems have specific capabilities that are essential for achieving uniformity. The test request and results are to be incorporated into a standard Health Level 7 (HL7) electronic format for laboratory test messaging. More information about HL7 can be found at http://www.hl7.org.

LOINC provides for a common understanding of the medical procedure or process related to the specific assay, in this case the process of detecting the presence of influenza virus and the potential sub-typing of the detected influenza virus. The LOINC codes specified here describe the important information about the methodology employed by the assay; recovery and amplification of one or more RNA targets. Multiple LOINC codes are utilized to convey that the assay is composed of multiple components, i.e. it is a panel or a battery of subtests. In the case of the CDC Human Influenza Virus Real-Time RT-PCR Diagnostic Panel, the LOINC also provides for conveying that an interpretive test summary is appropriate.

SNOMED CT codes provide for unambiguous representation of the test results and allow the application of specific concepts such as “detected” or “positive” or the identification of detected organism names. Though not further defined in this document, SNOMED CT can also be used to provide for description of the type and source/ location of the specimen being tested or for conveying information about failures of the test procedure or the lack of adequate specimen.

Specific Recommendations for Standards-Based Electronic Data Exchange for Influenza

Laboratories can find more information regarding implementation of HL7 messaging for CDC Flu rRT-PCR Dx Panel, including applicable LOINC test codes and SNOMED result codes at http://www.cdc.gov/flu/professionals/diagnosis/rtpcr-test-kits.htm

Quality Control

- Quality control requirements must be performed in conformance with local, state, and federal regulations or accreditation requirements and the user's laboratory's standard quality control procedures. It is recommended that the user refer to CLSI document C24-A2, Statistical Quality Control for Quantitative Measurements: Principles and Definitions: [Approved Guideline-Second Edition] or other published guidelines for general quality control recommendations. For further guidance on appropriate quality control practices, refer to
42CFR 493.1202(c).
- Quality control procedures are intended to monitor reagent and assay performance.
- Test all positive controls prior to running diagnostic samples with each new kit lot to ensure all reagents and kit components are working properly.
- Good laboratory practice (cGLP) recommends including a positive extraction control in each nucleic acid isolation batch.
- HSC extraction control must proceed through nucleic acid isolation per batch of specimens to be tested.
- Always include a negative control (NTC), and the appropriate positive control (i.e PIPC) in each amplification and detection run.

Limitations

- All users, analysts, and any person reporting diagnostic results should be trained to perform this procedure by a competent instructor. They should demonstrate their ability to perform the test and interpret the results prior to performing the assay independently. CDC Influenza Division will limit the distribution of this device to only those users who have successfully completed training provided by CDC instructors or designees. This device is subject to a special control requiring that distribution be limited to laboratories with (i) experienced personnel who has training in standardized molecular testing procedures and expertise in viral diagnosis, and (ii) appropriate biosafety equipment and containment (21 CFR866.3332(b)(2)).
- Negative results do not preclude influenza virus infection and should not be used as the sole basis for treatment or other patient management decisions.
- A false negative result may occur if a specimen is improperly collected, transported or handled. False negative results may also occur if amplification inhibitors are present in the specimen or if inadequate numbers of organisms are present in the specimen. Children tend to shed virus more abundantly and for longer periods of time than adults. Therefore, testing specimens from adults will have lower sensitivity than testing specimens from children.

- Positive and negative predictive values are highly dependent on prevalence. False negative test results are more likely during peak activity when prevalence of disease is high. False positive test results are more likely during periods of low influenza activity when prevalence is moderate to low.
- The performance of the assay has not been established in individuals who received nasally administered influenza vaccine. Individuals who received nasally administered influenza A vaccine may have positive test results for up to three days after vaccination. http://www.cdc.gov/mmwr/preview/mmwrhtml/rr57e717a1.htm
- Do not use any reagent past the expiration date.
- Optimum specimen types and timing for peak viral levels during infections caused by a novel influenza A virus have not been determined. Collection of multiple specimens from the same patient may be necessary to detect the virus.
- If the virus mutates in the rRT-PCR target region, a specific novel influenza A virus may not be detected or may be detected less predictably. Inhibitors or other types of interference may produce a false negative result. An interference study evaluating the effect of common cold medications was not performed.
- Test performance can be affected because the epidemiology and pathology of disease caused by a specific novel influenza A virus is not fully known. For example, clinicians and laboratories may not know the optimum types of specimens to collect, and when during the course of infection these specimens are most likely to contain levels of virus that can be readily detected.
- Detection of viral RNA may not indicate the presence of infectious virus or that influenza is the causative agent for clinical symptoms.
- The performance of this test has not been established for monitoring treatment of influenza A or B infection.
- The performance of this test has not been established for screening of blood or blood product for the presence of influenza A or B.
- This test cannot rule out diseases caused by other bacterial or viral pathogens.

Expected values

The expected values are derived from the clinical studies performed during the 2006-2007 (K080570), 2009-2010 (K101564), and 2011-2012 influenza seasons.

During February 25, 2012, to May 19, 2012, World Health Organization and National Respiratory and Enteric Virus Surveillance System (NREVSS) collaborating laboratories in the United States tested 47,281 respiratory specimens for influenza viruses. Of these, 9,415 (19.9%) were positive:
85% of the positive specimens were positive for influenza A viruses and 15% were positive for influenza B viruses. Among the 5,071 influenza A viruses for which subtyping was performed, 3,680 (72.6%) were influenza A/H3 viruses and 1,391 (27.4%) were 2009 H1N1 influenza viruses.

From August 30, 2009, through March 27, 2010, World Health Organization (WHO) and National Respiratory and Enteric Virus Surveillance System (NREVSS) collaborating laboratories in the United States tested 422,648 specimens. Of these, 89,585 (21.1%) were positive: 89,298 (99.7%) were positive for influenza A and 287 (0.3%) were positive for influenza B. Among 66,978 influenza

A viruses for which subtyping was performed, almost all (66,589 [99.4%]) were 2009 H1N1 viruses. Of the 37,260 specimens reported during February 14 - March 27, 2010, a total of 2,020 (5.4%) tested positive for influenza, of which 1,999 (98.9%) were positive for influenza A and 21 (1.0%)
were positive for influenza B. Of the 1,510 influenza A viruses reported since mid-February for which subtyping was performed, almost all (1,506 [99.7%]) were 2009 H1N1 viruses. No seasonal influenza A (H1) viruses and only three influenza A (H3) viruses were reported. During February 14 - March 27, states in the Southeast (Alabama, Florida, Georgia, Kentucky, Mississippi, North Carolina, South Carolina, and Tennessee) accounted for approximately 55% of the influenza positives reported but only 20% of the specimens tested.

During October 1, 2006--May 19, 2007, World Health Organization and National Respiratory and Enteric Virus Surveillance System (NREVSS) collaborating laboratories in the United States tested 179,268 respiratory specimens for influenza viruses; 23,753 (13.2%) were positive. Of these, 18,817 (79.2%) were influenza A viruses and 4,936 (20.8%) were influenza B viruses. Among the influenza A viruses, 6,280 (33.4%) were subtyped; 3,912 (62.3%) were influenza A/H1 viruses and
2,368 (37.7%) were influenza A/H3 viruses (http://www.cdc.gov/mmwr/preview/mmwrhtml/mm5631a2.htm). In the rRT-PCR Flu Panel multi- center prospective clinical study during the 2006-2007 influenza season, the prevalence as determined by virus culture was as follows: influenza A/H1 (7.0%), influenza A/H3 (23.6%), and influenza B (9.9%).

Performance Characteristics

Clinical Performance

The performance characteristics of oligonucleotide primer and probe sets of the Influenza A/B Typing assay were evaluated in multiple clinical investigations and influenza seasons. The combined summary clinical data are presented as well as an additional clinical evaluation to demonstrate the performance of the panel with an alternate enzyme chemistry.

Prospective clinical evaluations were performed during the 2006-2007, 2007-2008, 2009-2010, and 2011-2012 influenza seasons at 4-8 U.S. public health laboratories. In each study, samples were obtained from specimens collected for routine influenza testing at each site from individuals who were symptomatic for influenza-like illness (ILI) and included both upper respiratory and lower respiratory specimen types. To supplement the number of rare clinical specimens containing H5N1 influenza virus grown cultures were utilized. Low prevalence subtypes during a particular season were also supplemented with retrospective samples from previous seasons. Reference methods for detection and identification of influenza A and B viruses and influenza A/H1 and A/H3 subtypes during the 2006-2007, 2007-2008, and 2009-2010 seasons were rapid culture (shell vial) followed by direct fluorescent antibody screening and identification. A prospective clinical evaluation was also performed during the 2011-2012 influenza season at 6 U.S. public health laboratories to evaluate the performance of an alternate enzyme system. In this study the comparator method was the CDC device with the original cleared enzyme system. The table below provides a summary of each year’s study population by age group and by specimen type.

	Clinical Study/Season			
Patient Age	2006-2007	2007-2008	2009-2010	2011-2012
0-16	68	148	685	434
17-54	221	65	779	328
≥ 55	79	26	398	215
Not Reported	49	84	39	25
Specimen Type				
NPS	415		1021	798
NPS/TS		38	99	37
NA		14	57	13
NW			462	24
TS		84	50	8
NS			170	113
Isolates	24	187		
Lower Resp			42	5

Prospective Study Results

Influenza A Comparison

Clinical Study	Specimen Type	# of Positives[1]	% Sensitivity (95% CI)	# of Negatives[1]	% Specificity (95% CI)
2006-2007	NPS, NS	142/143	99.3 (96.1 - 99.9)	251/272	92.3 (88.5 - 94.9)
2007-2008	TS	17/17	100.0 (81.6 - 100.0)	59/60	98.3 (91.1 - 99.7)
	NA	4/4	100.0 (51.0 - 100.0)	10/10	100.0 (72.2 - 100.0)
	NPS/TS	15/15	100.0 (56.6 - 100.0)	17/20	100.0 (88.6 - 100.0)
	Isolates	10/10	100.0 (72.2 - 100.0)	108/174	62.1 (54.7 - 68.9)
2009-2010	NA, NW	19/19	100.0 (83.2 - 100.0)	281/283	99.3 (97.5 - 99.8)
	NPS, NS	61/63	96.8 (89.1 - 99.1)	783/814	96.2 (94.6 - 97.3)
	NPS/TS	6/6	100.0 (61.0 - 100.0)	59/59	100.0 (93.9 - 100.0)
	BAL, BW, TA	5/6	83.3 (43.6 - 97.0)	20/24	83.3 (64.1 - 93.3)

[1]Proportion of actual positives or actual negatives correctly identified versus the comparator (virus culture)

Influenza B Comparison

Clinical Study	Specimen Type	# of Positives[1]	% Sensitivity (95% CI)	# of Negatives[1]	% Specificity (95% CI)
2006-2007	NPS, NS	40/41	97.6 (87.4 - 99.6)	367/374	98.1 (96.2 - 99.1)
2007-2008	TS	18/20	90.0 (69.9 – 97.2)	32/57	56.1 (43.3 – 68.2)
	NA	7/7	100.0 (64.6 - 100.0)	7/7	100.0 (64.6 - 100.0)
	NPS/TS	5/5	100.0 (56.6 - 100.0)	30/30	100.0 (88.6 - 100.0)
	Isolates	19/20	95.0 (76.4 – 99.1)	101/164	61.6 (54.0 - 68.7)

[1]Proportion of actual positives or actual negatives correctly identified versus the comparator (virus culture)

Retrospective Study Results

Influenza A Comparison

Clinical Study	Specimen Type	# of Positives[1]	% Positive Agreement (95% CI)
2009-2010	NA, NW	209/214	97.7 (94.6 – 99.0)
	NPS, NS	310/312	99.4 (97.7 – 99.8)
	NPS/TS	31/31	100.0 (89.0 – 100.0)
	BAL, BW, TA	10/10	100.0 (72.3 - 100.0)

[1]Proportion of actual positives correctly identified versus the comparator (virus culture)

Analytical Performance

Analytical Sensitivity – Limit of Detection (LOD)

Analytical sensitivity of InfA and InfB primer and probe sets was demonstrated by determining the LOD of each primer and probe set. The LOD for each primer and probe set was calculated to indicate the lowest detectable concentration of influenza virus (EID_{50}/mL) at which ≥ 95% of all replicates tested positive.

Influenza Virus Tested	Influenza Strain Designation	LOD (EID_{50}/ML)
A/H1N1	A/New Caledonia/20/1999	$10^{1.2}$
	A/Hawaii/15/2001	$10^{1.5}$
A/H3N2	A/New York/55/2004	$10^{2.2}$
	A/Wisconsin/67/2005	$10^{1.2}$
A/H5N1	A/Vietnam/1203/2004×A/Puerto Rico/8/34 reassortant (A/Vietnam/1203/2004 PR8- VNH5N1- PR8/CDC-RG)	10^{0}
	A/Anhui/01/2005×A/Puerto Rico/8/34 reassortant (A/Anhui/01/2005- PR8-IBCDC-RG5)	10^{0}
B	B/Florida/07/2004 (B/Victoria/2/87 genetic group)	10^{0}
	B/Ohio/01/2005 (B/Yamagata/16/88 genetic group)	$10^{0.5}$
A/H1pdm09	A/California/07/2009	$10^{1.6}$
	A/New York/18/2009	$10^{1.3}$

Analytical Reactivity (Inclusivity)

The analytical inclusivity of the InfA and InfB primer and probe sets was demonstrated by testing ten (10) influenza virus strains of seasonal A/H1N1, A/H3N2, A/H1pdm09, and Influenza B at low virus concentrations at or near the LOD. There were 24 influenza A/H5N1 strains tested retrospectively from diverse geographic locations from suspect positive cases received at CDC. The Influenza A/B Typing assay analytical inclusivity indicated 100% concordance with expected results for all primer and probe sets included in the device.

The 2010-2011 influenza season viral strains were detected by the Influenza A/B Typing assay as demonstrated by analytical testing of 49 original specimens received from US public health laboratories from the 2010-2011 season. They were 24 Influenza A/H3 (49%) and 25 Influenza B (51%). Eighteen

original lower respiratory specimens were received from US public health laboratories during the 2010-2011 influenza season from hospitalized patients or fatal cases. The Influenza A/B Typing assay detected influenza A/H3, A/H1pdm09, and influenza B in bronchoalveolar lavage, bronchial washes, tracheal aspirates, sputum, and lung tissue specimens, verifying detection of recent circulating influenza viruses in the lower respiratory tract.

Analytical Specificity - Exclusivity

Non-Influenza Human Respiratory Pathogens

Analytical specificity (cross-reactivity) was evaluated by testing InfA and InfB primer/probe sets with nucleic acids extracted from 36 organisms (16 viruses, 19 bacteria, and 1 yeast) representing common respiratory pathogens or flora commonly present in specimens collected from the nasopharynx region. Bacteria and yeast were tested at concentrations greater than or equal to10^6 cfu/ml with the exception of *Chlamydia pneumoniae* which was quantified by determining inclusion forming units and *Mycobacterium tuberculosis* where DNA was extracted from pure culture and quantified by spectrophotometry. Non-influenza respiratory viruses were tested at concentrations greater than 10^6 $TCID_{50}$/mL with the exception of human parainfluenza type 2 ($10^{3.1}$ $TCID_{50}$/ml due to difficulty generating a high titer virus stock in culture) and human parainfluenza type 1 where RNA was extracted from viral culture and quantified by spectrophotometry. Similarly, RNA was extracted from viral cultures of Coronaviruses CoV 229E and CoV OC43 and concentrations were determined by spectrophotometry.

Organism Tested			Reactivity	
Bacteria and Yeast	Strain	cfu / mL	InfA	Inf B
Bordetella pertussis	A639	$10^{8.3}$	-	-
Candida albicans	2001-21-196	$10^{8.8}$	-	-
Chlamydia pneumoniae[1]	TW183	40 IFU/mL	-	nd[2]
Corynebacterium diphtheriae	NA[3]	10^{10}	-	-
Escherichia coli	K12	$10^{9.6}$	-	-
Haemophilus influenzae	M15709	$10^{6.4}$	-	-
Lactobacillus plantarum	NA	$10^{8.8}$	-	-
Legionella pneumophila	NA	$10^{10.3}$	-	-
Moraxella catarrhalis	M15757	$10^{9.5}$	-	-
Mycobacterium tuberculosis[4]	BCG	0.25 ng /µL	-	-
Mycobacterium tuberculosis[4]	H37Rv	95 ng/ µL	-	nd
Mycoplasma pneumoniae	MI-29	$10^{7.7}$	-	-
Neisseria elongata	NA	$10^{8.6}$	-	-
Neisseria meningitidis	M2578	$10^{7.9}$	-	-
Pseudomonas aeruginosa	NA	$10^{10.5}$	-	-
Staphylococcus epidermidis	NA	$10^{10.5}$	-	-
Staphylococcus aureus	NA	$10^{10.7}$	-	-
Streptococcus pneumoniae	249-06 (Thailand)	$10^{6.6}$	-	-
Streptococcus pyogenes	7790-06	$10^{7.5}$	-	-
Streptococcus salivarius	SS1672	$10^{8.4}$	-	-
Viruses	Strain	log $TCID_{50}$/mL	InfA	Inf B
Enterovirus	Echo 6	$10^{6.9}$	-	-
Human Adenovirus, type 1	Ad.71	$10^{9.2}$	-	-
Human Adenovirus, type 7a	S-1058	$10^{7.1}$	-	-
Human Coronavirus virus[4]	OC43	50.4 ng /µL	-	-
Human Coronavirus virus[4]	299E	31.6 ng /µL	-	-
Human Rhinovirus A	1A	$10^{5.8}$	-	-
Human Parainfluenza 1 virus[4]	NA	3.0 ng/ µL	-	-
Human Parainfluenza 2 virus	Greer	$10^{3.1}$	-	-
Human Parainfluenza 3 virus	C-243	$10^{7.9}$	-	-
Respiratory Syncytial virus	CH93-18b	$10^{6.8}$	-	-
Herpes Simplex Virus	KOS	$10^{8.4}$	-	nd
Varicella-zoster Virus	AV92-3	$10^{4.4}$	-	nd
Epstein Barr Virus[4]	B95-8	1.7 ng/µL	-	nd
Measles Virus	Edmonston	$10^{5.2}$	-	nd
Mumps Virus	Enders	$10^{7.2}$	-	nd
Cytomegalovirus	AD-169	$10^{6.9}$	-	nd

[1] Organism quantified by Infectious Forming Units (IFU)
[2] nd = not determined
[3] NA = not applicable
[4]Organism quantified by spectrophotometry (ng/µL)

The study demonstrated that the primer and probe sets contained within the CDC Human Influenza Real-Time RT-PCR Diagnostic Panel did not cross-react with any of the non-influenza respiratory pathogens or commensal organisms and demonstrated 100% concordance with the expected results.

Exclusivity with Influenza Viruses with Pandemic Potential

To demonstrate the ability of the InfA primer and probe set to detect influenza A/H5 viruses with pandemic potential, 10 avian A/H5 influenza viruses that have been shown to infect humans were tested. The avian A/H5 influenza viruses were grown and harvested from allantoic/amniotic fluid from infected embryonated chicken eggs (ECE) and titered. Each virus was extracted using the Roche MagNA Pure Compact RNA Isolation kit, and the purified RNA was tested in triplicate.

Avian A/H5 Influenza Viruses Tested at High Virus Titer

Type	Virus	Clade	Titer (log EID_{50}/mL)	InfA
H5N1	A/Japanese white eye/Hong Kong/1038/2006	2.3.4	9.2	+ (3/3)
	A/Duck/Hunan/795/2002	2.1	9.9	+ (3/3)
	A/Chicken/Yunnan/1251/2003	1	9.3	+ (3/3)
	A/Common magpie/Hong Kong/645/2006	2.3.2	9.2	+ (3/3)
	A/Vietnam/1194/2004	1	9.3	+ (3/3)
	A/Egypt/321/2007	2.2	9.2	+ (3/3)
	A/Anhui/1/2005	2.3.4	9.3	+ (3/3)
	A/Chicken/India/NIV3487/2006	2.2	9.3	+ (3/3)
	A/Chicken/Vietnam/NCVD-016/2008	7	9.1	+ (3/3)
	A/Cambodia/R040505/2007	1	8.5	+ (3/3)

To demonstrate the ability of the InfA primer and probe set to detect potential pandemic influenza A viruses and exclusivity of the InfB primer and probe set, 15 non-human influenza viruses that have been shown to infect humans were tested. The non-human influenza viruses were propagated in ECE, grown to high titer, and harvested as described previously. Each virus was extracted using Roche MagNA Pure Compact RNA kit.

Animal Influenza Viruses Tested at High Virus Titer

Species	Virus	Type	Titer (log EID_{50}/mL)	InfA	InfB
EQUINE	A/equine/Ohio/01/2003	EQ-H3N8	7.5	+ (3/3)	-
SWINE	A/Maryland/12/1991	SW-H1N1	9.0	+ (3/3)	nd[1]
SWINE	A/Iowa/1/2006	SW-H1N1	9.0	+ (3/3)	nd
AVIAN	A/chicken/Pennsylvania/298101-4/2004	H2N2	9.5	+ (3/3)	-
CANINE	A/canine/Florida/43/2004	H3N8	7.2	+ (3/3)	-
AVIAN	A/chicken/Alabama/1975	H4N8	10.0	+ (3/3)	-
AVIAN	A/chucker/Minnesota/14591-7/1998	H5N2	7.2	+ (3/3)	-
AVIAN	A/duck/Singapore-Q/F119-3/1997	H5N3	8.5	+ (3/3)	-
AVIAN	A/duck/Pennsylvania/1969	H6N1	9.2	+ (3/3)	-
AVIAN	A/chicken/California/32213-1/2000	H6N2	9.1	+ (3/3)	-
AVIAN	A/chicken/New York/13237-6/1998	H6N8	10.0	+ (3/3)	-
AVIAN	A/mallard/NL/12/2000 IB-CDC-1	H7N7	9.5	+ (3/3)	-
AVIAN	A/turkey/Wisconsin/66	H9N2	9.7	+ (3/3)	nd
AVIAN	A/chicken/New Jersey/15906-9/1996	H11N1	6.5	+ (3/3)	nd
SWINE	A/swine/Wisconsin/125/1997	SW-H1N1	7.9	+ (3/3)	-

[1] nd = not determined

Influenza A/B Typing Assay Reproducibility/Precision

The reproducibility and precision of the Influenza A/B Typing assay were evaluated in two studies prior to the merger of the CDC Human Influenza Virus Real-Time RT-PCR Detection and Characterization Panel (K080570) and the CDC rRT-PCR 2009 A(H1N1)pdm Flu Panel (K101564).The first study utilized three separate laboratory sites in a collaborative bridging study with Applied Biosystems using the ABI 7500 Fast Real-time PCR instruments and SDS software version 1.4. Instrument functionality was validated at each site by AB prior to the reproducibility assessment. The panel of 9 simulated samples that were used included influenza A/H1N1, A/H3N2, A/H5N1 (reassortant), and Influenza B at two viral RNA concentrations each (a low viral RNA concentration near the assay lower LOD range and a 1:10 dilution of the same sample). The low viral RNA concentration generally was one log above the assay cutoff for all analytes, whereas the 1:10 dilution of the same sample approximated a sample at the cutoff or a “high negative” for the different analytes. Four RNA purification methods were used to evaluate reproducibility of the CDC rRT-PCR Flu Panel on the validated ABI 7500 Fast Real-time PCR instruments: the automated MagNA Pure LC RNA Isolation Kit III (Roche Applied Science), the manual QIAGEN RNeasy® RNA

extraction (QIAGEN Ltd.), the Roche MagNA Pure LC Automated Isolation System with Total RNA isolation method, and the QIAGEN QIAamp® Viral RNA manual extraction method. The manufacturer's instructions for use provided in the package insert were followed.

The panels and assay controls were tested at each site by 2 operators on 5 different days within a 10-day period. Each site tested one of the four extraction methods associated with this device. Simulated samples in the panel used in the reproducibility evaluation were:

- **Sample #1** Influenza A/H1N1 at low viral RNA titer range
- **Sample #2** Influenza A/H1N1 at a 1:10 dilution of sample 1
- **Sample #3** Influenza A/H3N2 at low viral RNA titer range
- **Sample #4** Influenza A/H3N2 at a 1:10 dilution of sample 3
- **Sample #5** Influenza A/H5N1 WT at low viral RNA titer range
- **Sample #6** Influenza A/H5N1 WT at a 1:10 dilution of sample 5
- **Sample #7** Influenza B Yamagata at low viral RNA titer range
- **Sample #8** Influenza B Yamagata at a 1:10 dilution of sample 7
- **Sample #9** Influenza Negative (Uninfected A549 cells)

Bridging Study Statistical Analysis of the CDC Human Influenza Virus Real-Time RT-PCR Detection and Characterization Panel (K080570) on the ABI 7500 Dx Fast Real-time PCR Instrument

Sample and Analyte Tested	Roche MagNA Pure TNA			Qiagen QIAamp® RNA			Qiagen RNeasy®			Roche MagNA Pure RNA			Total Agreement w/ Expected Results	95 % CI
	Agreement w/ expected Result	Avg. Ct	% CV	Agreement w/ expected Result	Avg. Ct	% CV	Agreement w/ expected Result	Avg. Ct	% CV	Agreement w/ expected Result	Avg. Ct	% CV		
Sample 1 (low) - **InfA**	10/10	33.73	3.12	10/10	32.24	5.65	10/10	34.34	1.94	10/10	31.55	3.69	40/40	91.2–100.0
Sample 1 (low) - **RNaseP**	10/10	28.62	4.00	10/10	30.08	5.63	10/10	27.67	3.28	10/10	28.22	3.13	40/40	91.2–100.0
Sample 2 (1:10 of Sample 1) - **InfA**	9/10	36.59	3.22	10/10	35.30	5.93	10/10	37.03	4.07	10/10	33.97	3.32	39/40	86.8 - 99.9
Sample 2 (1:10 of Sample 1) - **RNaseP**	10/10	29.51	3.80	10/10	31.13	9.58	10/10	28.46	2.47	10/10	28.65	2.12	40/40	91.2–100.0
Sample 3 (low) - **InfA**	10/10	35.39	3.74	10/10	33.32	7.09	10/10	34.01	7.16	10/10	32.29	1.82	40/40	91.2–100.0
Sample 3 (low) - **RNaseP**	10/10	29.17	5.32	10/10	30.02	5.33	10/10	28.30	3.05	10/10	28.50	3.34	40/40	91.2–100.0
Sample 4 (1:10 of Sample 3) - **InfA**	7/10	38.44	4.05	10/10	37.03	5.08	5/10	39.05	3.46	10/10	36.18	2.41	32/40	64.4 - 91.0
Sample 4 (1:10 of Sample 3) - **RNaseP**	10/10	29.75	4.32	10/10	31.11	7.89	10/10	28.56	4.60	10/10	28.68	3.35	40/40	91.2–100.0
Sample 5 (low) - **InfA**	10/10	33.68	2.70	10/10	31.90	6.66	10/10	33.07	3.14	10/10	30.80	1.74	40/40	91.2–100.0
Sample 5 (low) - **RNaseP**	10/10	29.44	4.05	10/10	30.16	7.18	10/10	27.97	4.84	10/10	28.46	2.78	40/40	91.2–100.0
Sample 6 (1:10 of Sample 5) - **InfA**	8/10	36.90	6.60	10/10	34.84	4.89	8/10	37.10	5.15	10/10	33.54	2.61	36/40	76.3 – 97.2
Sample 6 (1:10 of Sample 5) - **RNaseP**	10/10	30.05	3.58	10/10	30.12	3.39	10/10	28.59	4.37	10/10	28.66	3.45	40/40	91.2–100.0
Sample 7 (low) - **Inf B**	8/10	34.10	4.25	10/10	33.49	9.12	10/10	34.70	7.70	10/10	32.83	3.61	38/40	83.1 – 99.4
Sample 7 (low) - **RNaseP**	10/10	29.78	3.55	10/10	30.75	10.16	10/10	28.54	4.42	10/10	28.90	3.13	40/40	91.2–100.0
Sample 8 (1:10 of Sample 7) - **Inf B**	1/10	36.76	ND*	8/10	37.1	5.77	3/10	38.95	ND*	8/10	37.11	5.81	20/40	33.8 – 66.2
Sample 8 (1:10 of Sample 7) - **RNaseP**	10/10	29.48	4.46	10/10	30.58	6.30	10/10	28.71	3.35	10/10	28.92	4.07	40/40	91.2–100.0
Sample 9 Influenza (-) **RNaseP**	10/10	29.79	3.17	10/10	30.08	5.91	10/10	28.27	4.83	10/10	28.86	3.68	40/40	91.2–100.0

Notes: * The CV% is not determined because more than 5 of the samples were negative and the analyte was not detected

A second reproducibility and precision study was conducted with the CDC rRT-PCR 2009 A(H1N1)pdm Flu Panel (K101564) at six separate laboratory sites. Each testing site assessed a panel of four simulated samples at relative moderate, low (near the assay lower LOD range), and "high negative" viral RNA concentration, and a negative sample. The panels and assay controls were tested at each site by two operators on five different days within a 10-day period. Each site tested one of the six extractions methods associated with this device. Simulated samples in the qualification panel used in the reproducibility evaluation were:

Sample #1 Influenza 2009 A/H1N1 moderate viral RNA titer range
Sample #2 Influenza 2009 A/H1N1 low viral RNA titer range
Sample #3 Influenza 2009 A/H1N1 "high negative" RNA titer range
Sample #4 Influenza Negative (uninfected A549 cells)

Summary of Reproducibility Study for the CDC rRT-PCR 2009 A(H1N1)pdm Flu Panel (K101564)

N=10 tests		bioMerieux NucliSENS easyMAG			Roche MagNA Pure LC TNA			Roche MagNA Pure Compact NA			Qiagen QIAcube Viral RNA			Qiagen Manual Viral RNA			Roche MagNA Pure Compact RNA				
		Agreement with expected result	Avg. Ct	% CV	Agreement with expected result	Avg. Ct	% CV	Agreement with expected result	Avg. Ct	% CV	Agreement with expected result	Avg. Ct	% CV	Agreement with expected result	Avg. Ct	% CV	Agreement with expected result	Avg. Ct	% CV	**Total Agreement**	**95% CI**
**No Template Control**		10/10	NA	NA	10/10	NA	NA	10/10	NA	NA	10/10	NA	NA	10/10	NA	NA	10/10	NA	NA	**60/60**	**94.0-100**
**Sample 1 moderate**	**InfA**	10/10	28.52	2.59	10/10	30.53	2.55	10/10	31.99	4.23	10/10	27.92	2.06	10/10	28.85	2.00	10/10	30.54	2.12	**60/60**	**94.0-100**
	**RP**	10/10	28.78	1.33	10/10	30.79	2.65	10/10	32.27	2.51	10/10	27.55	2.43	10/10	27.82	2.45	10/10	29.43	1.77	**60/60**	**94.0-100**
**Sample 2 low**	**InfA**	10/10	33.10	3.26	8/10	35.28	5.54	8/10	36.12	4.11	10/10	32.95	1.04	10/10	33.49	3.14	10/10	34.48	2.88	**58/60**	**88.5-99.6**
	**RP**	10/10	28.44	1.34	10/10	30.33	2.78	10/10	31.44	3.27	10/10	27.41	1.95	10/10	28.34	1.55	10/10	28.57	5.11	**60/60**	**94.0-100**
**Sample 3 "high negative"**	**InfA**	8/10	NA	NA	10/10	NA	NA	9/10	NA	NA	10/10	NA	NA	7/10	NA	NA	10/10	NA	NA	**54/60**	**79.5-96.2**
	**RP**	10/10	28.04	2.13	10/10	30.08	4.92	10/10	31.12	2.60	10/10	26.89	2.09	10/10	28.33	1.41	10/10	28.45	3.31	**60/60**	**94.0-100**
**Sample 4 A549 cells**	**RP**	10/10	28.68	2.75	10/10	30.20	5.68	10/10	32.10	1.74	10/10	27.06	2.65	10/10	28.60	2.29	10/10	29.33	4.00	**60/60**	**94.0-100**
**HSC**	**RP**	10/10	28.51	2.30	10/10	29.80	4.48	10/10	29.55	3.11	10/10	25.89	2.04	10/10	27.30	1.61	10/10	27.35	4.98	**60/60**	**94.0-100**
**Influnza 2009 A(H1N1) pdm Positive Control**	**InfA**	10/10	23.71	2.10	10/10	21.95	2.97	10/10	23.54	4.10	10/10	21.62	1.39	10/10	23.66	2.63	10/10	24.95	2.21	**60/60**	**94.0-100**
	**RP**	10/10	28.84	1.66	10/10	27.44	3.23	10/10	29.03	4.03	10/10	27.35	1.84	10/10	28.59	2.35	10/10	28.82	3.26	**60/60**	**94.0-100**

Enzyme Equivalency

Clinical Comparison

A prospective clinical study was conducted during the 2011-2012 influenza season to compare the performance of the CDC Human Influenza rRT-PCR Diagnostic Panel using Quanta BioSciences qScript™ One-Step qRT-PCR Kit, Low ROX (Quanta qScript™) and Invitrogen Superscript™ III Platinum® One-Step Quantitative RT-PCR Kit (Invitrogen Superscript™). Residual material from a total of 1,002 respiratory specimens from patients who were symptomatic for influenza-like illness (ILI) was collected and tested at 6 clinical sites. Nine hundred thirty-one specimens were included in the data analysis after exclusion of samples with inconclusive results (42), technician or instrument error (25), or unspecified specimen type (4). The results from the prospective study are summarized in the table below showing the percent sensitivity or specificity with the two-sided 95% confidence interval.

Prospective Study Comparison

Assay Result	# of Positives[1]	% Positive Agreement (95% CI)	# of Negatives[1]	% Negative Agreement (95% CI)
InfB	77/80	96.3 (89.5 – 98.7)	851/851	100.0 (99.6 – 100.0)
A/H1	0	NA[2]	931/931	100.0 (99.6 – 100.0)
A/H3	331/335	98.8 (97.0 – 99.5)	595/596	99.8 (99.1 – 100.0)
A/H1pdm09	43/43	100.0 (91.8 – 100.0)	888/888	100.0 (99.6 – 100.0)

[1]Proportion of actual positives or actual negatives correctly identified versus the comparator
[2]NA = not applicable

Due to the absence of seasonal influenza A/H1N1, retrospective specimens from a previous clinical study conducted during the 2006-2007 influenza season were used to supplement the clinical evaluation. The results of the retrospective specimen testing are summarized in the table below.

A/H1 Retrospective Study Results

Assay Result	# of Positives[1]	% Positive Agreement (95% CI)	# of Negatives[1]	% Negative Agreement (95% CI)
A/H1	30/30	100.0 (88.7 – 100.0)	0	NA[2]

[1]Proportion of actual positives or actual negatives correctly identified versus the comparator
[2]NA = not applicable

Due to the lack of available clinical specimens containing influenza A/H5N1, evaluation of the performance of the H5a and H5b primer and probe sets was addressed with an alternative approach using contrived samples of stock virus added to an A549 cell suspension in high, moderate, and low concentrations. The results are summarized in the tables below.

A/H5 Retrospective Study Results

A/H5 Comparison High Virus Concentration

		Invitrogen SuperScript™			
		Positive	Negative	Inconclusive	Total
Quanta qScript™	Positive	12	0	0	12
	Negative	0	0	0	0
	Inconclusive	0	0	0	0
	Total	12	0	0	12

A/H5 Comparison Moderate Virus Concentration

		Invitrogen SuperScript™			
		Positive	Negative	Inconclusive	Total
Quanta qScript™	Positive	12	0	0	12
	Negative	0	0	0	0
	Inconclusive	0	0	0	0
	Total	12	0	0	12

A/H5 Comparison Low Virus Concentration

		Invitrogen SuperScript™			
		Positive	Negative	Inconclusive	Total
Quanta qScript™	Positive	1	0	1	2
	Negative	0	0	0	0
	Inconclusive	2	0	8	10
	Total	3	0	9	12

Fifty negative specimens were obtained from a clinical study conducted during the 2006-2007 influenza season and tested with the A/H5 assay from the CDC Human Influenza rRT-PCR Diagnostic Panel using Quanta qScript™ and Invitrogen Superscript™. The results with the negative specimens showed 100% agreement with a 95% confidence interval of 92.9-100.0.

Analytical Reactivity and Sensitivity

Analytical sensitivity was demonstrated by determining the LOD of each primer and probe set in the CDC Human Influenza Virus Real-time RT-PCR Diagnostic Panel using Quanta qScript™ to demonstrate equivalency to the LOD using Invitrogen SuperScript™.The LOD was calculated to indicate the range of the lowest detectable concentration of influenza virus (EID_{50} /mL or $TCID_{50}$ /mL) at which ≥95% of all replicates tested positive. The lowest concentration of influenza virus detected determined the end-point concentration where both the type and subtype primer and probe sets had uniform detection. If the two endpoints differed in concentration, the lowest concentration where the

endpoints had uniform detection was reported as the LOD. Results are summarized in the table below.

LOD Summary Comparison Table

Influenza Virus Type/Subtype	Influenza Virus	LOD (EID_{50}/mL)	
		Invitrogen SuperScript™	Quanta qScript™
A/H1N1	A/Brisbane/59/2007	$10^{2.3}$	$10^{2.3}$
	A/Fujian Gulou/1896/2009	$10^{2.7}$	$10^{2.7}$
A/H1pdm09	A/California/07/2009	$10^{1.6}$	$10^{3.0}$
	A/South Carolina/2/2010	$10^{2.1}$	$10^{2.8}$
A/H3N2	A/Perth/16/2009	$10^{2.8}$	$10^{2.8}$
	A/Victoria/361/2011	$10^{2.8}$	$10^{2.8}$
A/H5N1	A/Vietnam/1203/2004-PR8/CDC-RG	$10^{1.2}$	$10^{1.2}$
	A/Anhui/01/2005-PR8-IBCDC-RG6	$10^{1.7}$	$10^{2.4}$
B	B/Wisconsin/01/2010	$10^{2.1}$	$10^{2.8}$
	B/Nevada/01/2011	$10^{1.4}$	$10^{0.7}$

The analytical and clinical performance of the CDC Human Influenza rRT-PCR Diagnostic Panel using Quanta qScript™ is substantially equivalent when compared with the Invitrogen Superscript™. Both enzyme kits are cleared for use with the CDC Human Influenza rRT-PCR Diagnostic Panel.

Disposal

Dispose of hazardous or biologically contaminated materials according to the practices of your institution.

References

1. Ballew, H. C., *et al*. “Basic Laboratory Methods in Virology,” DHHS, Public Health Service 1975 (Revised 1981), Centers for Disease Control and Prevention, Atlanta, Georgia 30333.

2. Calfee *et al.* "Safety and Efficacy of Intravenous Zanamivir in Preventing Experimental Human Influenza A Virus Infection,” *Antimicrobial Agents in Chemotherapy,* July 1999; 43(7), 1616–1620.

3. Clinical Laboratory Standards Institute (CLSI), “Collection, Transport, Preparation and Storage of Specimens for Molecular Methods: Proposed Guideline,” MM13-P, Vol. 25, No. 9.

4. Clinical Laboratory Standards Institute (CLSI), “User Protocol for Evaluation of Qualitative Test Performance," Guideline EP-12A.

5. Clinical Laboratory Standards Institute (CLSI), “Genotyping for Infectious Disease: Identification and Characterization; Approved Guideline,’ MM10-A, Vol. 26, No. 9.

6. Doller *et al*. "Direct Detection of Influenza Virus Antigen in Nasopharyngeal Specimens by Direct Enzyme Immunoassay in Comparison with Quantitating Virus Shedding," *Journal of Clinical Microbiology*, April 1992, 866-869.

7. Giard, D. J., *et al*. "*In vitro* Cultivation of Human Tumors: Establishment of Cell Lines Derived from a Series of Solid Tumors." *Journal of the National Cancer Institute* 1973, 51(5), 1417-23.

8. Hall, H. "Standard Operating Procedure BPP/052 (Inoculation of Embryonated Chicken Eggs)," DHHS, Public Health Service 1982, Centers for Disease Control and Prevention, Atlanta, Georgia 30333.

9. Kaiser *et al*. "Performance of Virus Isolation and Directigen® Flu A to Detect Influenza A Virus in Experimental Human Infection," *Journal of Clinical Virology* 1999, 14, 191–197.

10. Lieber, M., *et al*. "A Continuous Tumor Cell Line from a Human Lung Carcinoma with Properties of Type II Alveolar Epithelial Cells." *International Journal of Cancer* 1976, 17(1), 62-70.

11. Ng *et al*. "Influenza A H5N1 Detection," *Emerging Infectious Diseases*, August 2005,11(8), 1303-1305.

12. Peiris, J. S. M. *et al*. "Re-emergence of Fatal Human Influenza A Subtype H5N1 Disease. *Lancet* 2004, 363, 617-619.

13. Reed, L. J. and H. A. Muench. "Simple Method of Estimating Fifty Percent Endpoints." *American Journal of Hygiene* 1938, 27, 493-497.

14. Ryan-Poirier *et al*. "Application of Directigen FLU-A for the Detection of Influenza A Virus in Human and Nonhuman Specimens," *Journal of Clinical Microbiology*, May 1992, 30(5), 1072-1075.

15. Snyder *et al*. "Evaluation of Live Avian-Human Reassortant Influenza A H3N2 and HINI Virus Vaccines in Seronegative Adult Volunteers," *Journal of Clinical Microbiology*, May 1986, 852-857.

16. Szretter, K. J., *et al*. " Influenza: Propagation, Quantification, and Storage" in Current Protocols in Microbiology 2006 15G.1.1-15G.1.22; Editors: R. Coica, T. Kowalik, J. M. Quarles, B. Stevenson, R. K. Taylor, A. E. Simon and T. Downey; John Wiley & Sons, Inc. Hoboken, NJ.

17. United States Department of Health and Human Services. "Laboratory Diagnostics. Supplement 2 to *HHS Pandemic Influenza Plan,*" Washington D.C, November 2005, [cited 12 January 2006], http://www.hhs.gov/pandemicflu/plan/

18. United States Food and Drug Administration, "Content of a 510(k)," http://www.fda.gov/cdrh/devadvice/314312.htmlmlmlml#link_10

19. United States Food and Drug Administration, “Statistical Guidance on Reporting Results from Studies Evaluating Diagnostic Test,” http://www.fda.gov/cdrh/osb/guidance/1620.htmlmlmlml

20. White L. A. “Standard Operating Procedure BPP/041 (Innocuity Testing of Viral Reagents),” DHHS, Public Health Service 1982, Centers for Disease Control and Prevention, Atlanta, Georgia 30333.

21. World Health Organization, “Guidelines for the Collection of Clinical Specimens During Field Investigation of Outbreaks,” http://www.who.int/csr/resources/publications/surveillance/whocdscsredc2004.pdf

22. World Health Organization, “Avian Influenza, Including Influenza A (H5N1), in Humans: WHO Interim Infection Control Guideline for Health Care Facilities,” http://www.who.int/csr/disease/avian_influenza/guidelines/infectioncontrol1/en/

Contact Information, Ordering, and Product Support

For technical and product support, contact the CDC Influenza Division Support team directly.

For Technical Support:

Log onto the FluSupport SharePoint site at

https://partner.cdc.gov/Sites/NCIRD/FS/default.aspx

Or

Send email to: FluSupport@cdc.gov

For Ordering:

FluOrder@cdc.gov

Or

Log onto influenzareagentresource.org
